# Postsurgery Subjective Cognitive and Short-Term Memory Impairment Among Middle-Aged Chinese Patients

**DOI:** 10.1001/jamanetworkopen.2023.36985

**Published:** 2023-10-10

**Authors:** Lei Yang, Wenwen Chen, Di Yang, Dongxu Chen, Yuanyuan Qu, Yao Hu, Di Liu, Junhui He, Yuling Tang, Huolin Zeng, Haiyang Li, Yuyang Zhang, Zi Ye, Jin Liu, Qian Li, Huan Song

**Affiliations:** 1 Department of Anesthesiology and West China Biomedical Big Data Center, West China Hospital, Sichuan University, Chengdu, China; 2 West China Biomedical Big Data Center, West China Hospital, Sichuan University, Chengdu, China; 3 Med-X Center for Informatics, Sichuan University, Chengdu, China; 4 Department of Anesthesiology, West China Hospital, Sichuan University, Chengdu, China; 5 Department of Anesthesiology, Sichuan Academy of Medical Science and Sichuan Provincial People’s Hospital, University of Electronic Science and Technology of China, Chengdu, China; 6 Sichuan University–Pittsburgh Institute, Sichuan University, Chengdu, China; 7 Center of Public Health Sciences, Faculty of Medicine, University of Iceland, Reykjavík, Iceland; 8 Institute of Environmental Medicine, Karolinska Institute, Stockholm, Sweden

## Abstract

**Importance:**

Perioperative neurocognitive disorder, particularly postoperative cognitive impairment, is common and associated with multiple medical and social adversities, although data from China are lacking.

**Objective:**

To examine the incidence, trajectory, and risk factors for subjective cognitive and short-term memory impairment after surgery in the Chinese population.

**Design, Setting, and Participants:**

This cohort study used data from the China Surgery and Anesthesia Cohort to assess surgical patients aged 40 to 65 years from 2 medical centers between July 15, 2020, and March 31, 2023, with active follow-up within 1 year after the surgery. Of 11 158 patients who were successfully recruited (response rate, 94.4%), 10 149 participants were eligible and available for analysis. From this population, separate cohorts were constructed for analyzing subjective cognitive impairment (8105 noncardiac and 678 cardiac surgery patients) and short-term memory impairment (5246 noncardiac and 454 cardiac surgery patients).

**Exposures:**

Twenty-four potential risk factors regarding comorbidities, preoperative psychological conditions, anesthesia- or surgery-related factors, and postsurgical events were included.

**Main Outcomes and Measures:**

Outcomes included subjective cognitive function measured by the 8-Item Informant Interview to Differentiate Aging and Dementia (AD8; scores range from 0 to 8, with higher scores indicating more severe cognitive impairment) and short-term memory measured by the 3-Word Recall Test (TRT; scores range from 0 to 3, with lower scores indicating more severe short-term memory impairment) at 1, 3, 6, and 12 months after noncardiac and cardiac surgery. Generalized linear mixed models were used to identify risk factors associated with the presence of AD8 (score ≥2) or TRT (score <3) abnormality as well as the aggressively deteriorative trajectories of those cognitive measurements.

**Results:**

For noncardiac surgery patients, the AD8 analysis included 8105 patients (mean [SD] age, 52.3 [7.1] years; 3378 [41.7%] male), and the TRT analysis included 5246 patients (mean [SD] age, 51.4 [7.0] years; 1969 [37.5%] male). The AD8 abnormality incidence rates after noncardiac surgery increased from 2.2% (175 of 8105) at 7 days to 17.1% (1059 of 6191) at 6 months after surgery, before appearing to decrease. In contrast, the TRT abnormality incidence rates followed a U-shaped pattern, with the most pronounced incidence rates seen at 7 days (38.9% [2040 of 5246]) and 12 months (49.0% [1394 of 2845]). Similar patterns were seen among cardiac surgery patients for the AD8 analysis (678 patients; mean [SD] age, 53.2 [6.3] years; 393 [58.0%] male) and TRT analysis (454 patients; mean [SD] age, 52.4 [6.4] years; 248 [54.6%] male). Among noncardiac surgery patients, the top risk factors for aggressively deteriorative AD8 trajectory and for AD8 abnormality, respectively, after surgery were preoperative sleep disturbances (Pittsburgh Sleep Quality Index ≥16 vs 0-5: odds ratios [ORs], 4.04 [95% CI, 2.20-7.40] and 4.54 [95% CI, 2.40-8.59]), intensive care unit stay of 2 days or longer (ORs, 2.43 [95% CI, 1.26-4.67] and 3.07 [95% CI, 1.67-5.65]), and preoperative depressive symptoms (ORs, 1.76 [95% CI, 1.38-2.24] and 2.23 [95% CI, 1.79-2.77]). Analyses for TRT abnormality and trajectory, as well as the analyses conducted among cardiac surgery patients, found fewer associated factors.

**Conclusions and Relevance:**

This cohort study of middle-aged Chinese surgery patients found subjective cognitive and short-term memory impairment within 12 months after both cardiac and noncardiac surgery, with multiple identified risk factors, underscoring the potential of preoperative psychological interventions and optimized perioperative management for postoperative cognitive impairment prevention.

## Key Points

**Question:**

What are the incidence, trajectory, and influential factors of subjective cognitive and short-term memory impairment after surgery in the Chinese population?

**Findings:**

This cohort study of 10 149 middle-aged Chinese people found subjective cognitive and short-term memory impairment within 12 months after cardiac and noncardiac surgery. Some patients experienced an aggressively deteriorative trajectory of subjective cognitive impairment after surgery, with possible pronounced risk factors that included preoperative sleep disturbances, intensive care unit stay of 2 days or longer, and preoperative depressive symptoms.

**Meaning:**

These findings highlight the potential for preoperative psychological interventions and optimized perioperative management to prevent postoperative cognitive impairment.

## Teaser

This cohort study of middle-aged Chinese people examines the incidence and risk factors of subjective cognitive and short-term memory impairment after surgery and identifies major patterns of cognitive trajectories.

## Introduction

Postoperative cognitive dysfunction or impairment,^1^ as a particular format and important component of the broader concept of perioperative neurocognitive disorder,^2^ is a common complication of surgery and anesthesia. With large variations by age and surgery type, the incidence rates of postoperative cognitive dysfunction are most marked in older patients after cardiac surgery (16.0%-39.0% at 3 months),^3,4^ whereas the rates after major noncardiac surgery (9.9%-12.7%) or surgery in younger patients (5.6% of those aged 40-59 years)^5,6^ are lower. On the basis of small studies, a comparable incidence of postoperative cognitive impairment was reported among older Chinese surgery patients (10.3%-18.2%).^7,8^ However, data from middle-aged Chinese adults with cognitive function measured up to 12 months after surgery are lacking. In addition to direct impairments in perception, thinking, and memory ability, cognitive impairment has been found to be associated with increased medical costs after surgery,^9^ dismissal from work due to disabilities,^10^ and even increased premature deaths.^6^ Therefore, understanding the cognitive trajectories of surgery patients and identifying risk factors, particularly the modifiable ones that might contribute to postoperative cognitive deterioration, are crucial and have great clinical importance in terms of developing interventions for postoperative cognitive impairment prevention.

Despite mixed results, prior reviews^11,12^ have summarized factors relevant to the risk of postoperative cognitive dysfunction, such as demographic characteristics (eg, age and educational level), disease history (eg, vascular diseases and diabetes), anesthesia- and surgery-related factors (eg, major surgery, intraoperative bleeding, and cerebral oxygen desaturation), and postoperative events (eg, postoperative delirium and pain). Most of those studies have limitations, including small sample sizes,^13^ high rates of loss to follow-up,^14^ and insufficient follow-up periods to explore time-specific cognitive impairment (especially after discharge).^15,16^ Consequently, the approach to identify patients at high risk of postoperative cognitive impairment and the underlying mechanisms of such a condition remain largely unclear.

Leveraging data from the China Surgery and Anesthesia Cohort (CSAC), which prospectively collected enriched multidimensional information on lifestyles, perioperative somatic and psychological conditions, as well as multiple surgery- and anesthesia-related factors and outcomes from more than 10 000 Chinese surgical patients aged 40 to 65 years, we conducted a cohort study to (1) describe the incidence of subjective cognitive and short-term memory impairment measured at different postoperative time points and identify major patterns of cognitive trajectories within 1 year after surgery and (2) explore potential risk factors associated with the presence or the aggressively deteriorative trajectory of subjective cognitive and short-term memory impairment. With stringent quality control processes to guarantee the accuracy of the obtained data and the large sample size, our study has the potential to significantly advance our knowledge about postoperative cognitive impairment and its influential factors among the middle-aged Chinese population.

## Methods

### Study Design

The CSAC is an ongoing multicenter cohort study recruiting patients aged 40 to 65 years who underwent elective surgery and general anesthesia in 4 medical centers in China (3 hospitals in Sichuan and 1 in Hebei Province) since July 15, 2020. Details of the CSAC have been reported elsewhere^17^ (eMethods and eFigure 1 in Supplement 1). Until March 31, 2023, a total of 11 814 eligible surgery patients were invited to participate, among whom 11 158 were successfully recruited into this study (response rate, 94.4%). Written informed consent was obtained from all participants before baseline data collection. The CSAC and the present study were approved by the Biomedical Research Ethics Committee of West China Hospital, Sichuan University. This study followed the Strengthening the Reporting of Observational Studies in Epidemiology (STROBE) reporting guideline.^18^

The present study was based on the CSAC database, from which we identified all eligible patients recruited from 2 medical centers (West China Hospital and West China Tianfu Hospital) between July 15, 2020, and March 31, 2023. Specifically, based on a total of 9411 patients who underwent noncardiac surgery, we excluded participants who had more than 1 surgery (n = 78); from the remaining 9333 participants, we constructed 2 separate cohorts for analyzing subjective cognitive impairment presented as 8-Item Informant Interview to Differentiate Aging and Dementia (AD8) abnormality (score ≥2) and short-term memory impairment presented as 3-Word recall test (TRT) abnormality (score <3) (described in the Measurement of Postoperative Cognitive Function section). Within this noncardiac surgery cohort, 8105 participants were analyzed for postoperative AD8 abnormality (after excluding those 228 with missing AD8 scores and 1000 with a baseline AD8 score ≥2). Likewise, 5246 noncardiac patients were analyzed for TRT abnormality (after excluding 594 with missing TRT scores and 3493 a baseline TRT score less than 3) (eFigure 2 in Supplement 1).

Following a similar population selection procedure, from 829 enrolled patients who received cardiac surgery, 13 were excluded for receiving more than 1 surgery; from the remaining 816 available patients, we analyzed 678 for AD8 abnormality and 454 TRT abnormality (eFigure 2 in Supplement 1). The follow-up rates within 12 months (ie, the proportion of participants having either AD8 or TRT score at each follow-up) were 94.0% (4682 of 4982) to 98.8% (9133 of 9241) after noncardiac surgery and 94.2% (419 of 445) to 97.6% (789 of 808) after cardiac surgery.

#### Measurement of Postoperative Cognitive Function

We applied both subjective and objective tools to measure postoperative cognitive function. Specifically, the AD8^19^ was used to measure self-rated changes (yes or no) in cognitive performance using 8 questions covering 4 cognition-related domains (ie, memory, problem-solving, orientation, and daily activities) at 7 days and 1, 6, and 12 months after the surgery (ie, any change since the date of the surgery) (eTable 1 in Supplement 1). Possible scores range from 0 to 8, with a higher score indicating potentially more severe subjective cognitive impairment. We considered an AD8 score of 2 or greater to represent significant AD8 abnormality, indicating notable cognitive impairment.^19^ As a brief and quick questionnaire for general screening, the AD8 was found to have a high correlation with clinical neurologic assessments^20^ and other screening tools (eg, Montreal Cognitive Assessment [MoCA]^21^) in validation studies, and it has been widely used in large-scale cohort studies of both the general population and surgery patients.^22,23^

The TRT, which asks participants to recall 3 unrelated words shortly after they are stated (usually with several short questions in between), was used to objectively measure short-term memory as an important aspect of cognitive function. The test was conducted at baseline, 7 days, and 1, 3, 6, and 12 months after the surgery. We used the Word Recall part of the Mini-Cog^24^ and applied different words listed in the Three Word Registration for each survey. The TRT is considered a sensitive screening tool for amnesia among individuals with mild cognitive impairment.^25^ Possible scores range from 0 to 3, with a lower score indicating potentially more severe short-term memory impairment; we defined a score less than 3 as TRT abnormality, indicating short-term memory impairment.

#### Potential Risk Factors and Covariates

The potential risk factors for interest (n = 24) included preoperative comorbidities, psychological conditions, anesthesia- or surgery-related factors, and postsurgical events (see measurement approaches and data sources in eTable 2 in Supplement 1). Data on sociodemographic (age, sex, ethnicity, educational attainment, and occupation) and lifestyle (body mass index [calculated as weight in kilograms divided by height in meters squared], smoking, and drinking) factors were collected through face-to-face interviews at baseline. Data on ethnicity were collected to provide information about the generalizability of the study.

### Statistical Analysis

We performed separate analyses for patients who underwent noncardiac surgery and those who underwent cardiac surgery. To illustrate the changes in postoperative cognitive function, we plotted mean AD8 and TRT scores as well as the percentages of postoperative AD8 and TRT abnormality at different follow-up time points. Furthermore, participants with a minimum of 2 measurements of postoperative cognitive function were included to identify the major trajectories of changes in cognitive function up to 12 months after surgery. We applied latent class trajectory models^26^ with second-order polynomials to identify the latent classes of individuals following similar progression patterns over time.

To examine the associations of each potential risk factor (ie, the 24 different items listed in eTable 2 in Supplement 1) with postoperative AD8 and TRT abnormality (as binary variables), as well as the aggressively deteriorative trajectory of postoperative cognitive function manifested by changes of AD8 or TRT scores, we used generalized linear mixed models with random intercepts to each participant, adjusted for age at baseline (as a continuous variable), sex (male or female), ethnicity (Han or others [including Tibetan, Mongol, Manchu, Qiang, Tujia, Hui, Zhuang, Bai, Bouyei, Kelso, and Dai]), educational attainment (middle school or lower, high school, junior college, or college or above), occupation (blue collar, white collar, self-employed, retired, or other), body mass index (<18.5, 18.5-24.9, 25-29.9, or ≥30), smoking status (yes or no) and drinking status (yes or no), and site (head and neck, thorax, abdomen, limbs and surface, or other) and type (endoscopic or open) of surgery. In addition to evaluating all the aforementioned factors separately (fitting 1 multivariate model for each factor), we constructed a mutually adjusted model to take into account the possible confounding effects among those factors.

We further performed subgroup analyses by age (40-55 and 56-65 years) and sex (male and female), with differences between subgroups tested by introducing an interaction term. We also conducted a sensitivity analysis in which the risk factors for the aggressively deteriorative trajectory were identified using individuals with the consistently low-risk trajectory instead of individuals clustered in both consistently low-risk and recovering trajectories as the reference group. All analyses were performed with R software, version 4.0 (R Foundation for Statistical Computing). A 2-sided *P* < .05 was considered statistically significant.

## Results

A total of 10 149 participants were eligible and available for analysis. Among 8105 participants who received noncardiac surgery and were included in the cohort for AD8 abnormality analysis, the mean (SD) age was 52.3 (7.1) years; 3378 (41.7%) were male and 4727 (58.3%) were female (Table 1). Similarly, the mean (SD) age was 51.4 (7.0) years for the 5246 participants who underwent noncardiac surgery and were included in the analytic cohort for TRT abnormality; this group also had characteristics comparable to those analyzed for AD8 abnormality, including a predominance of females (3277 [62.5%] vs 1969 [37.5%] males). In contrast, participants who underwent cardiac surgery (678 in the AD8 analysis and 454 in the TRT analysis) were more likely to be male (393 [58.0%] vs 285 [42.0%] female in the AD8 group and 248 [54.6%] vs 206 [45.4%] female in the TRT group) and were generally older (mean [SD] age, 53.2 [6.3] years in the AD8 group and 52.4 [6.4%] in the TRT group). Compared with noncardiac surgery patients, cardiac surgery patients generally had a lower socioeconomic status and had a higher likelihood of having unfavorable lifestyles and experiencing severe anesthesia- or surgery-related events (eg, intraoperative blood transfusion, severe hypoxia, severe hypotension, and postoperative complications) (Table 1).

**Table 1.** Basic Characteristics of the Study Population^a^

Characteristic	Participants receiving noncardiac surgery		Participants receiving cardiac surgery	
	Analytic cohort for AD8 abnormality (AD8 ≥2, n = 8105)	Analytic cohort for TRT abnormality (TRT <3, n = 5246)	Analytic cohort for AD8 abnormality (AD8 ≥2, n = 678)	Analytic cohort for TRT abnormality (TRT <3, n = 454)
Study centers				
West China Hospital	7196 (88.8)	4744 (90.4)	NA	NA
West China Tianfu Hospital	909 (11.2)	502 (9.6)	678 (100)	454 (100)
Social demographic data				
Sex				
Male	3378 (41.7)	1969 (37.5)	393 (58.0)	248 (54.6)
Female	4727 (58.3)	3277 (62.5)	285 (42.0)	206 (45.4)
Age, mean (SD), y	52.3 (7.1)	51.4 (7.0)	53.2 (6.3)	52.4 (6.4)
Ethnicity				
Han	8026 (99.0)	5194 (99.0)	653 (96.3)	434 (95.6)
Other^b^	79 (1.0)	52 (1.0)	25 (3.7)	20 (4.4)
Educational attainment				
Middle school and lower	2094 (25.8)	1030 (19.6)	347 (51.2)	198 (43.6)
High school	1805 (22.3)	1088 (20.7)	142 (20.9)	109 (24.0)
Junior college	1765 (21.8)	1275 (24.3)	94 (13.9)	72 (15.9)
College and above	2441 (30.1)	1853 (35.3)	95 (14.0)	75 (16.5)
Occupation				
Blue collar	652 (8.0)	308 (5.9)	157 (23.2)	80 (17.6)
White collar	3395 (41.9)	2476 (47.2)	174 (25.7)	140 (30.8)
Self-employed	1111 (13.7)	674 (12.8)	77 (11.4)	58 (12.8)
Retired	2361 (29.1)	1446 (27.6)	168 (24.8)	115 (25.3)
Other^c^	579 (7.1)	338 (6.4)	102 (15.0)	61 (13.4)
Missing	7 (0.1)	4 (0.01)	NA	NA
Lifestyle factors				
BMI				
<18.5	288 (3.6)	206 (3.9)	25 (3.7)	19 (4.2)
18.5-24.9	5550 (68.5)	3670 (70.0)	409 (60.3)	275 (60.6)
25-29.9	2049 (25.3)	1243 (23.7)	218 (32.2)	144 (31.7)
≥30	218 (2.7)	127 (2.4)	26 (3.8)	16 (3.5)
Smoking^d^	2134 (26.3)	1201 (22.9)	250 (36.9)	152 (33.5)
Drinking^e^	1556 (19.2)	924 (17.6)	187 (27.6)	119 (26.2)
Preoperative comorbidities				
Psychiatric disorder	131 (1.6)	96 (1.8)	6 (0.9)	7 (1.5)
Diabetes	570 (7.0)	317 (6.0)	47 (6.9)	29 (6.4)
Hepatic disease	958 (11.8)	640 (12.2)	85 (12.5)	58 (12.8)
Peptic ulcer	356 (4.4)	215 (4.1)	42 (6.2)	24 (5.3)
Solid tumor	334 (4.1)	210 (4.0)	2 (0.3)	2 (0.4)
Chronic pain^f^	1437 (17.7)	997 (19.0)	117 (17.3)	88 (19.4)
Pain in the week before surgery	626 (7.7)	418 (8.0)	95 (14.0)	67 (14.8)
Psychological condition				
Anxiety (GAD-7 score ≥5)	443 (5.5)	385 (7.3)	39 (5.8)	28 (6.2)
Depression (PHQ-9 score ≥5)	667 (8.2)	553 (10.5)	67 (9.9)	55 (12.1)
Sleep disturbance (PSQI score)				
0-5	4366 (53.9)	2749 (52.4)	305 (45.0)	201 (44.3)
6-10	2894 (35.7)	1921 (36.6)	275 (40.6)	187 (41.2)
11-15	785 (9.7)	532 (10.1)	87 (12.8)	59 (13.0)
≥16	60 (0.7)	44 (0.8)	11 (1.6)	7 (1.5)
Anesthesia- and surgery-related factors				
Site of surgery				
Head and neck	1714 (21.2)	1124 (21.4)	NA	NA
Thorax	1335 (16.5)	910 (17.4)	678 (100)	454 (100)
Abdomen	3430 (42.3)	2152 (41.0)	NA	NA
Limbs and surface	1158 (14.3)	799 (15.2)	NA	NA
Others	468 (5.8)	261 (5.0)	NA	NA
Type of surgery				
Endoscopic surgery	4148 (51.2)	2725 (51.9)	26 (3.8)	19 (4.2)
Open surgery	3957 (48.8)	2521 (48.1)	652 (96.2)	435 (95.8)
Anesthesia duration, h^g^				
≤3/6	5360 (66.1)	3615 (68.9)	533 (78.6)	364 (80.2)
>3/6	1779 (22.0)	1093 (20.8)	137 (20.2)	84 (18.5)
Missing	966 (11.9)	538 (10.3)	8 (1.2)	6 (1.3)
ASA grade				
<3	7388 (91.2)	4822 (91.9)	19 (2.8)	12 (2.6)
≥3	717 (8.8)	424 (8.1)	659 (97.2)	442 (97.4)
Type of general anesthesia maintenance				
Total intravenous anesthesia	368 (4.5)	234 (4.5)	32 (4.7)	20 (4.4)
Combined intravenous and inhalation anesthesia	7654 (94.4)	4951 (94.4)	638 (94.1)	428 (94.3)
Inhalation anesthesia	81 (1.0)	59 (1.1)	8 (1.2)	6 (1.32)
Missing	2 (0.02)	2 (0.04)		
Combined with nerve block	2738 (33.8)	1710 (32.6)	33 (4.9)	24 (5.3)
Intraoperative blood transfusion	289 (3.6)	164 (3.1)	565 (83.3)	372 (81.9)
Severe hypoxia^h^	448 (5.5)	270 (5.2)	153 (22.6)	98 (21.6)
Severe hypotension^i^	1509 (18.6)	986 (18.8)	567 (83.6)	373 (82.2)
Postsurgical events				
Patient-controlled analgesia	3272 (40.4)	2041 (38.9)	14 (2.1)	13 (2.9)
Admission to ICU	164 (2.0)	93 (1.8)	646 (95.3)	428 (94.3)
Length of ICU stay, d				
0	7816 (96.4)	5079 (96.8)	27 (4.0)	20 (4.4)
1	82 (1.0)	50 (1.0)	117 (17.3)	87 (19.2)
≥2	73 (0.9)	37 (0.7)	518 (76.4)	333 (73.4)
Missing	134 (1.7)	80 (1.5)	16 (2.1)	14 (3.1)
Acute postoperative pain (BPI ≥4 at 3 d)	352 (4.3)	196 (3.7)	35 (5.2)	25 (5.5)
Any postoperative complications^j^	890 (11.0)	518 (9.9)	406 (59.9)	261 (57.5)

Abbreviations: AD8, 8-Item Informant Interview to Differentiate Aging and Dementia; ASA, American Society of Anesthesiologists; BMI, body mass index (calculated as weight in kilograms divided by height in meters squared); BPI, Brief Pain Inventory; GAD-7, Generalized Anxiety Disorder 7-item; ICU, intensive care unit; NA, not applicable; PHQ-9, Patient Health Questionnaire-9; PSQI, Pittsburgh Sleep Quality Index; TRT, 3-Word Recall Test.

^a^ Data are presented as number (percentage) of participants unless otherwise indicated.

^b^ Other ethnicities included Tibetan, Mongol, Manchu, Qiang, Tujia, Hui, Zhuang, Bai, Bouyei, Kelao, and Dai.

^c^ Other included unemployed and unspecified occupation.

^d^ Smoking at least 1 cigarette every 3 days for half a year at any time during the patient’s life.

^e^ Drinking at least once a week for half a year at any time during the patient’s life.

^f^ Pain duration of more than 1 month.

^g^ Anesthesia duration was categorized into 3 hours or less and more than 3 hours for noncardiac surgery and 6 hours or less and more than 6 hours for cardiac surgery.

^h^ Oxygen saturation less than 90% during the surgery.

^i^ Atrial pressure less than 60% of baseline level during the surgery.

^j^ Included pulmonary complication, major adverse cardiac event, acute kidney injury, and infection.

**Figure 1.**
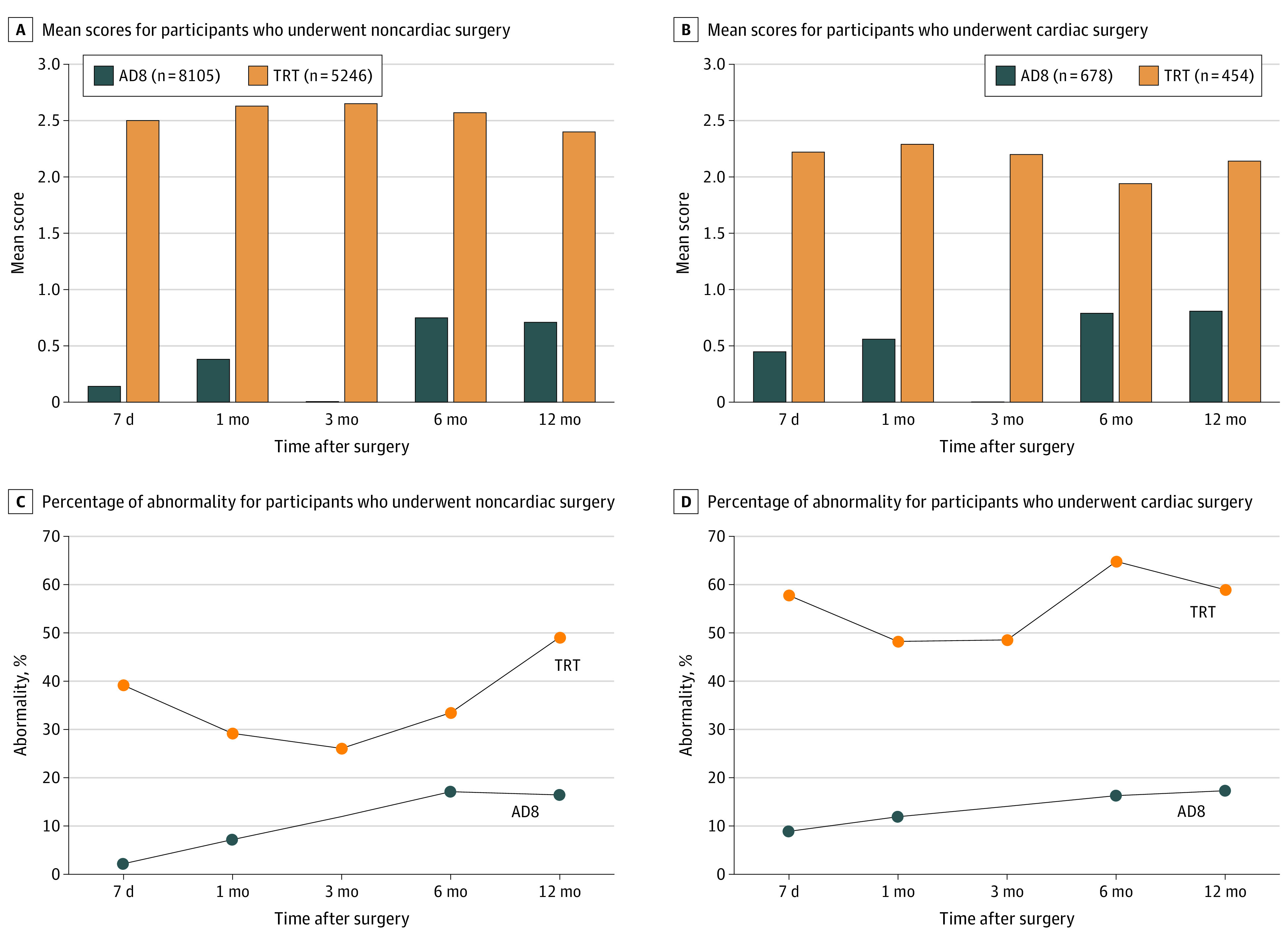
Mean Scores of the 8-Item Informant Interview to Differentiate Aging and Dementia (AD8) and 3-Word Recall Test (TRT) and Percentages of AD8 and TRT Abnormality at Different Follow-Up Times Among Participants Undergoing Noncardiac and Cardiac Surgery AD8 scores range from 0 to 8, with a higher score indicating potentially more severe subjective cognitive impairment. TRT scores range from 0 to 3, with a lower score indicating potentially more severe short-term memory impairment. AD8 abnormality (AD8 score ≥2) indicates subjective cognitive impairment, and TRT abnormality (TRT score <3) indicates short-term memory impairment.

When plotting the changes in AD8 scores (Figure 1A) and the incidence of AD8 abnormality (Figure 1B) within 12 months after noncardiac surgery, we observed a clear upward trend within 6 months, which seemed to decrease afterward. Specifically, the percentage of AD8 abnormality increased from 2.2% (175 of 8105) at 7 days to 17.1% (1059 of 6191) at 6 months after the surgery, which then remained stable (16.4% [722 of 4399]) at the 12-month follow-up. With generally higher point estimates, we obtained similar changing patterns of AD8 scores and AD8 abnormality incidence after cardiac surgery (Figure 1C and D). In contrast, changing patterns of TRT scores and the incidence of TRT abnormality indicated a notable decline in short-term memory 7 days after both noncardiac surgery (Figure 1A and B) and cardiac surgery (Figure 1C and D), followed by a recovering curve within 3 months after the index surgery (eg, decreased from 38.9% at 7 days to 26.0% at 3 months after noncardiac surgery and from 57.7% at 7 days to 48.5% at 3 months after cardiac surgery). However, the impairment worsened again during 3 to 12 months of follow-up to a level similar as or even inferior to that immediately after the surgery (eg, 49.0% and 58.9% at 12 months after noncardiac and cardiac surgery, respectively).

**Figure 2.**
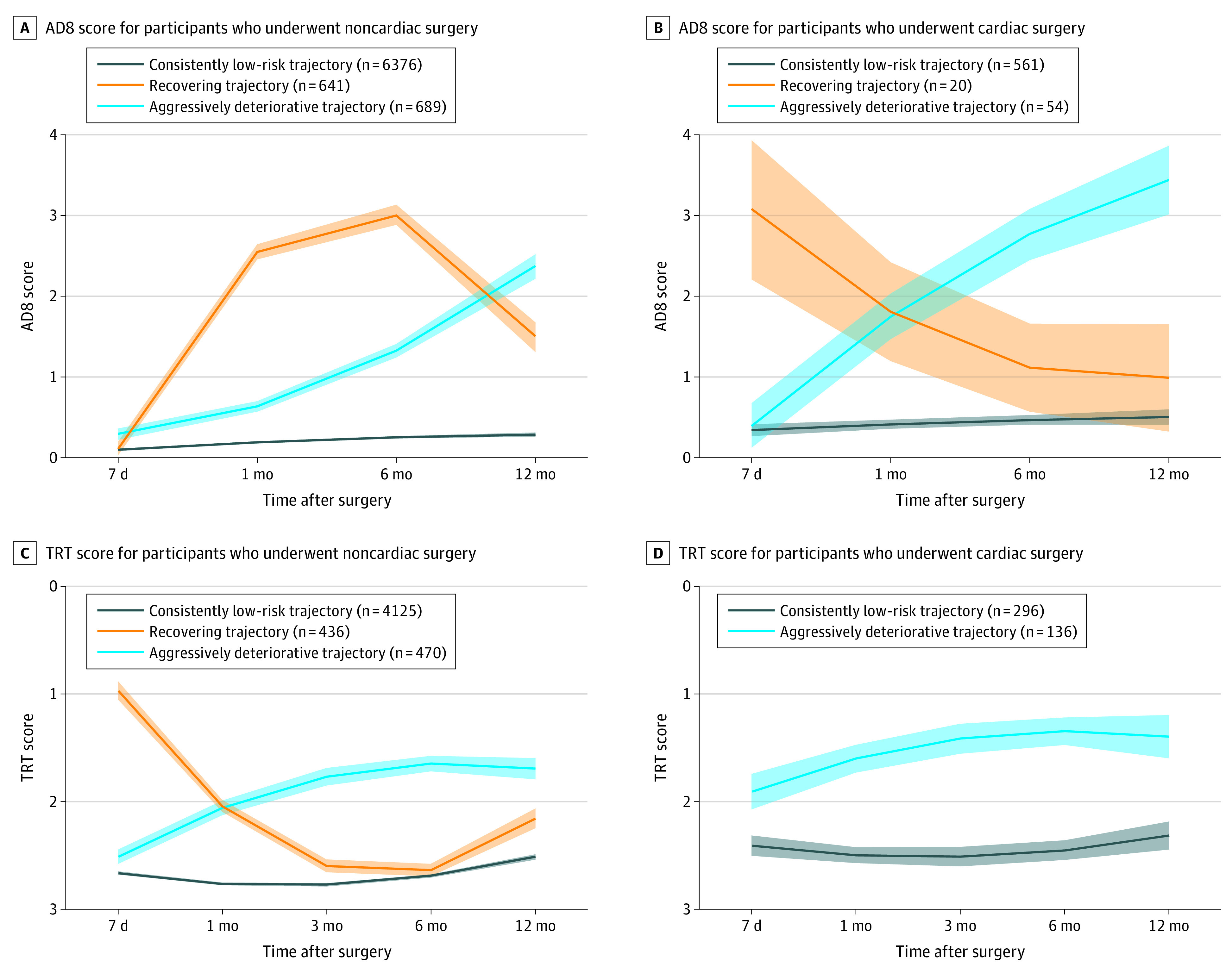
Trajectories of the 8-Item Informant Interview to Differentiate Aging and Dementia (AD8) and 3-Word Recall Test (TRT) Score Within 12 Months After Surgery AD8 scores range from 0 to 8, with a higher score indicating potentially more severe subjective cognitive impairment. TRT scores range from 0 to 3, with a lower score indicating potentially more severe short-term memory impairment. Shading around the lines represents confidence intervals for the calculated trajectory. We changed the direction of the y-axis (ie, with decreasing values from bottom to top) to facilitate the comparations between AD8 and TRT trajectories.

Among participants who underwent noncardiac surgery, a trajectory analysis of the AD8 scores (as continuous variables) was conducted with 7706 of 8105 participants (95.1%), and the analysis of TRT scores (as continuous variables) had 5031 of 5246 participants (95.9%). For the AD8 scores, we identified 3 trajectories (Figure 2A and eFigure 3A in Supplement 1), which were labeled according to their visualized changing patterns as (1) a consistently low-risk trajectory, with a mean (SD) score of 0.10 (0.33) at 7 days after surgery that then kept stable over time (n = 6376); (2) a recovering trajectory, with a mean (SD) score that increased from 0.33 (0.89) at 7 days to 3.75 (2.09) at 6 months followed by a recovering curve (n = 641); and (3) an aggressively deteriorative trajectory, with a mean (SD) score that increased from 0.42 (0.85) at 7 days after surgery to 2.49 (1.27) at 12 months (n = 689). Similarly, we determined 3 identical changing patterns of trajectories (ie, consistently low-risk, recovering, and aggressively deteriorative trajectories) for TRT scores after noncardiac surgery (Figure 2C and eFigure 3B in Supplement 1) as well as for the AD8 scores and TRT scores after cardiac surgery (Figure 2B and D; eFigure 3C and D in Supplement 1).

After adjusting for baseline sociodemographic and lifestyle factors, our analyses identified 10 risk factors associated with the aggressively deteriorative trajectory of AD8 scores and 13 risk factors associated with the occurrence of AD8 abnormality (Table 2) after noncardiac surgery, with the most pronounced odds ratios (ORs) noted for preoperative sleep disturbance (Pittsburgh Sleep Quality Index ≥16 vs 0-5: ORs, 4.04 [95% CI, 2.20-7.40] and 4.54 [95% CI, 2.40-8.59], respectively), intensive care unit (ICU) stay of 2 days or longer (ORs, 2.43 [95% CI, 1.26-4.67] and 3.07 [95% CI, 1.67-5.65], respectively), preoperative depressive symptoms (ORs, 1.76 [95% CI, 1.38-2.24] and 2.23 [95% CI, 1.79-2.77], respectively), admission to ICU (ORs, 1.81 [95% CI, 1.10-2.97] and 2.12 [95% CI, 1.38-3.26], respectively), psychiatric disorders (ORs, 1.95 [95% CI, 1.19-3.19] and 1.69 [95% CI, 1.03-2.77], respectively), and pain in the week before surgery (ORs, 1.45 [95% CI, 1.11-1.89] and 1.57 [95% CI, 1.57-1.57], respectively). Furthermore, in addition to receiving general anesthesia for more than 3 hours (ORs, 1.32 [95% CI, 1.10-1.59] and 1.47 [95% CI, 1.25-1.71], respectively) and having open surgery (ORs, 1.31 [95% CI, 1.08-1.58] and 1.27 [95% CI, 1.09-1.49], respectively), which were associated with both AD8 abnormality and the aggressively deteriorative trajectory of AD8 scores, other anesthesia- and surgery-related factors, such as site of surgery (eg, ORs 1.38 [95% CI, 1.01-1.89] for thorax and 1.68 [95% CI, 1.32-2.15] for abdomen), seemed to be important for the unfavorable AD8 trajectory only (Table 2). Eleven risk factors for TRT abnormality and 1 for the aggressively deteriorative trajectory of TRT scores after noncardiac surgery were identified (Table 2). In brief, we found lower but similar estimates for preoperative sleep disturbance and depressive symptoms as well as certain postsurgical events, whereas significant associations were additionally noted between some intraoperative conditions (ie, intraoperative blood transfusion and severe hypoxia) and the presence of TRT abnormality. The analyses conducted among participants who underwent cardiac surgery also suggested the importance of some preoperative psychological conditions on AD8 abnormality, whereas null results were found for most anesthesia- and surgery-related factors and TRT abnormality (Table 3).

**Table 2.** Associations of Studied Factors With the Occurrence or Aggressively Deteriorative Trajectory of Subjective Cognitive or Short-Term Memory Impairment Following Noncardiac Surgery

Variable	Postoperative AD8 abnormality (AD8 ≥2, n = 8105)		Aggressively deteriorative trajectory of AD8 scores (n = 7706)		Postoperative TRT abnormality (TRT <3, n = 5246)			Aggressively deteriorative trajectory of TRT scores (n = 5031)	
	No./total No. (%) of cases	OR (95% CI)^a^	No./total No. (%) of cases	OR (95% CI)^b^	No./total No. (%) of cases	OR (95% CI)^a^	No./total No. (%) of cases		OR (95% CI)^b^
Preoperative comorbidities									
Psychiatric disorder									
No	1825/7974 (22.9)	1.0 [Reference]	669/7585 (8.8)	1.0 [Reference]	3878/5150 (75.3)	1.0 [Reference]	460/4940 (9.3)		1.0 [Reference]
Yes	39/131 (29.8)	1.69 (1.03-2.77)	20/121 (16.5)	1.95 (1.19-3.19)	67/96 (69.8)	1.02 (0.78-1.34)	10/91 (11.0)		1.50 (0.75-2.98)
Diabetes									
No	1747/7535 (23.2)	1.0 [Reference]	641/7171 (8.9)	1.0 [Reference]	3687/4929 (74.8)	1.0 [Reference]	437/4728 (9.2)		1.0 [Reference]
Yes	117/570 (20.5)	0.87 (0.66-1.14)	48/535 (9.0)	1.04 (0.76-1.43)	258/317 (81.4)	1.09 (0.94-1.27)	33/303 (10.9)		0.92 (0.62-1.36)
Hepatic disease									
No	1610/7147 (22.5)	1.0 [Reference]	592/6783 (8.7)	1.0 [Reference]	3429/4606 (74.4)	1.0 [Reference]	404/4404 (9.2)		1.0 [Reference]
Yes	254/958 (26.5)	1.20 (0.99-1.46)	97/923 (10.5)	1.25 (0.99-1.57)	516/640 (80.6)	1.06 (0.95-1.18)	66/627 (10.5)		1.03 (0.77-1.37)
Peptic ulcer									
No	1779/7749 (23.0)	1.0 [Reference]	656/7362 (8.9)	1.0 [Reference]	3774/5031 (75.0)	1.0 [Reference]	444/4821 (9.2)		1.0 [Reference]
Yes	85/356 (23.9)	1.27 (0.92-1.76)	33/344 (9.6)	1.20 (0.82-1.74)	171/215 (79.5)	1.35 (1.13-1.61)	26/210 (12.4)		1.45 (0.93-2.25)
Solid tumor									
No	1776/7771 (22.8)	1.0 [Reference]	653/7391 (8.8)	1.0 [Reference]	3784/5036 (75.1)	1.0 [Reference]	450/4830 (9.3)		1.0 [Reference]
Yes	88/334 (26.4)	1.23 (0.90-1.69)	36/315 (11.4)	1.21 (0.84-1.74)	161/210 (76.7)	1.09 (0.91-1.30)	20/201 (10.0)		1.11 (0.68-1.81)
Chronic pain^c^									
No	1352/6276 (21.5)	1.0 [Reference]	500/5968 (8.4)	1.0 [Reference]	3150/4188 (75.2)	1.0 [Reference]	367/4022 (9.1)		1.0 [Reference]
Yes	379/1437 (26.4)	1.50 (1.27-1.78)	140/1358 (10.3)	1.21 (0.99-1.47)	751/997 (75.3)	1.04 (0.95-1.14)	98/948 (10.3)		1.12 (0.88-1.43)
Pain in the week before surgery									
No	1542/7087 (21.8)	1.0 [Reference]	566/6730 (8.4)	1.0 [Reference]	3573/4767 (75.0)	1.0 [Reference]	433/4569 (9.5)		1.0 [Reference]
Yes	189/626 (30.2)	1.57 (1.57-1.57)	74/596 (12.4)	1.45 (1.11-1.89)	328/418 (78.5)	1.07 (0.94-1.22)	32/401 (8.0)		0.81 (0.55-1.19)
Preoperative psychological condition									
Anxiety (GAD-7 score)									
<5	1733/7662 (22.6)	1.0 [Reference]	642/7292 (8.8)	1.0 [Reference]	3667/4861 (75.4)	1.0 [Reference]	438/4661 (9.4)		1.0 [Reference]
≥5	131/443 (29.6)	1.95 (1.48-2.57)	47/414 (11.4)	1.35 (0.98-1.87)	278/385 (72.2)	1.10 (0.96-1.27)	32/370 (8.6)		1.38 (0.93-2.05)
Depression (PHQ-9 score)									
<5	1639/7438 (22.0)	1.0 [Reference]	596/7079 (8.4)	1.0 [Reference]	3520/4693 (75.0)	1.0 [Reference]	422/4505 (9.4)		1.0 [Reference]
≥5	225/667 (33.7)	2.23 (1.79-2.77)	93/627 (14.8)	1.76 (1.38-2.24)	425/553 (76.8)	1.17 (1.04-1.31)	48/526 (9.1)		1.07 (0.77-1.48)
Sleep disturbance (PSQI score)									
0-5	794/4366 (18.2)	1.0 [Reference]	284/4154 (6.8)	1.0 [Reference]	1996/2749 (72.6)	1.0 [Reference]	206/2643 (7.8)		1.0 [Reference]
6-10	774/2894 (26.7)	1.66 (1.44-1.91)	284/2751 (10.3)	1.43 (1.20-1.70)	1487/1921 (77.4)	1.19 (1.11-1.29)	187/1843 (10.2)		1.20 (0.97-1.49)
11-15	265/785 (33.8)	2.49 (2.01-3.09)	105/744 (14.1)	1.87 (1.46-2.39)	424/532 (79.7)	1.30 (1.15-1.47)	77/503 (15.3)		1.85 (1.37-2.49)
≥16	31/60 (51.7)	4.54 (2.40-8.59)	16/57 (28.1)	4.04 (2.20-7.40)	38/44 (86.4)	1.34 (0.92-1.97)	0/42 (0)		NA
Anesthesia- or surgery-related factors									
Site of surgery									
Head and neck	385/1714 (22.5)	1.0 [Reference]	111/1640 (6.8)	1.0 [Reference]	825/1124 (73.4)	1.0 [Reference]	100/1077 (9.3)		1.0 [Reference]
Thorax	296/1335 (22.2)	0.97 (0.76-1.24)	105/1293 (8.1)	1.38 (1.01-1.89)	699/910 (76.8)	0.98 (0.86-1.11)	64/890 (7.2)		0.73 (0.50-1.06)
Abdomen	808/3430 (23.6)	1.14 (0.94-1.38)	327/3254 (10.0)	1.68 (1.32-2.15)	1647/2152 (76.5)	1.07 (0.96-1.18)	223/2064 (10.8)		0.94 (0.71-1.24)
Limbs and surface	305/1158 (26.3)	1.00 (0.80-1.25)	127/1096 (11.6)	1.47 (1.12-1.93)	575/799 (72.0)	0.96 (0.85-1.08)	63/762 (8.3)		0.76 (0.54-1.08)
Other	70/468 (15.0)	0.62 (0.43-0.89)	19/423 (4.5)	0.62 (0.37-1.03)	199/261 (76.2)	1.19 (0.99-1.43)	20/238 (8.4)		0.66 (0.39-1.12)
Type of surgery									
Endoscopic surgery	879/4148 (21.2)	1.0 [Reference]	323/3637 (8.9)	1.0 [Reference]	2030/2725 (74.5)	1.0 [Reference]	228/2629 (8.7)		1.0 [Reference]
Open surgery	985/3957 (24.9)	1.27 (1.09-1.49)	366/3746 (9.8)	1.31 (1.08-1.58)	1915/2521 (76.0)	1.05 (0.98-1.13)	242/2402 (10.1)		1.06 (0.84-1.33)
Anesthesia duration, h									
≤3	1267/5360 (23.6)	1.0 [Reference]	466/5183 (9.0)	1.0 [Reference]	2703/3615 (74.8)	1.0 [Reference]	318/3511 (9.1)		1.0 [Reference]
>3	515/1779 (29.0)	1.47 (1.25-1.71)	206/1713 (12.0)	1.32 (1.10-1.59)	831/1093 (76.0)	1.12 (1.02-1.22)	119/1060 (11.2)		1.11 (0.87-1.41)
ASA grade									
<3	1698/7388 (23.0)	1.0 [Reference]	623/7041 (8.8)	1.0 [Reference]	3589/4822 (74.4)	1.0 [Reference]	410/4627 (8.9)		1.0 [Reference]
≥3	166/717 (23.2)	0.98 (0.77-1.23)	66/665 (9.9)	1.14 (0.86-1.50)	356/424 (84.0)	1.18 (1.03-1.34)	60/404 (14.8)		1.31 (0.96-1.79)
Type of general anesthesia maintenance									
Total intravenous anesthesia	106/368 (28.8)	1.0 [Reference]	36/357 (10.1)	1.0 [Reference]	175/234 (74.8)	1.0 [Reference]	25/227 (11.0)		1.0 [Reference]
Combined intravenous and inhalation anesthesia	1738/7654 (22.7)	0.85 (0.63-1.14)	638/7267 (8.8)	0.81 (0.56-1.16)	3724/4951 (75.2)	1.08 (0.91-1.28)	435/4744 (9.2)		0.80 (0.51-1.26)
Inhalation anesthesia	20/81 (24.7)	0.84 (0.42-1.65)	15/80 (18.8)	1.90 (0.97-3.73)	44/59 (74.6)	1.08 (0.75-1.55)	9/58 (15.5)		1.49 (0.62-3.56)
Combined with nerve block									
No	1203/5367 (22.4)	1.0 [Reference]	436/5085 (8.6)	1.0 [Reference]	2628/3536 (74.3)	1.0 [Reference]	294/3380 (8.7)		1.0 [Reference]
Yes	661/2738 (24.1)	1.06 (0.92-1.23)	253/2621 (9.6)	1.07 (0.90-1.28)	1317/1710 (77.0)	1.01 (0.93-1.10)	176/1651 (10.7)		1.24 (0.99-1.54)
Intraoperative blood transfusion									
No	1776/7682 (23.1)	1.0 [Reference]	661/7430 (8.9)	1.0 [Reference]	3764/5002 (75.2)	1.0 [Reference]	451/4877 (9.2)		1.0 [Reference]
Yes	88/289 (30.4)	1.31 (0.94-1.83)	28/274 (10.2)	0.98 (0.65-1.48)	139/164 (84.8)	1.36 (1.11-1.66)	19/153 (12.4)		0.99 (0.59-1.65)
Severe hypoxia^d^									
No	1704/6698 (25.4)	1.0 [Reference]	639/6480 (9.9)	1.0 [Reference]	3324/4440 (74.9)	1.0 [Reference]	404/4317 (9.4)		1.0 [Reference]
Yes	79/448 (17.6)	0.88 (0.65-1.20)	33/422 (7.8)	0.75 (0.52-1.09)	211/270 (78.2)	1.20 (1.02-1.41)	33/256 (12.9)		1.31 (0.88-1.95)
Severe hypotension^e^									
No	1415/5633 (25.1)	1.0 [Reference]	535/5447 (9.8)	1.0 [Reference]	2793/3723 (75.0)	1.0 [Reference]	336/3621 (9.3)		1.0 [Reference]
Yes	368/1509 (24.4)	0.90 (0.76-1.07)	137/1452 (9.4)	0.90 (0.74-1.11)	742/986 (75.2)	1.01 (0.92-1.11)	101/951 (10.6)		1.08 (0.85-1.38)
Postsurgical events									
Patient-controlled analgesia									
No	1077/4833 (22.3)	1.0 [Reference]	384/4583 (8.4)	1.0 [Reference]	2347/3205 (73.2)	1.0 [Reference]	270/3072 (8.8)		1.0 [Reference]
Yes	787/3272 (24.0)	1.29 (1.08-1.52)	305/3123 (9.8)	1.23 (1.01-1.50)	1598/2041 (78.3)	1.14 (1.04-1.25)	200/1959 (10.2)		1.26 (0.98-1.61)
Admission to ICU									
No	1811/7807 (23.2)	1.0 [Reference]	669/7547 (8.9)	1.0 [Reference]	3826/5073 (75.4)	1.0 [Reference]	458/4941 (9.3)		
Yes	53/164 (32.3)	2.12 (1.38-3.26)	20/157 (12.7)	1.81 (1.10-2.97)	77/93 (82.8)	1.35 (1.03-1.76)	12/89 (13.5)		1.43 (0.75-2.75)
Length of ICU stay, d									
0	1815/7816 (23.2)	1.0 [Reference]	670/7556 (8.9)	1.0 [Reference]	3832/5079 (75.4)	1.0 [Reference]	460/4947 (9.3)		1.0 [Reference]
1	17/82 (20.7)	0.97 (0.50-1.89)	7/81 (8.6)	1.29 (0.58-2.86)	40/50 (80.0)	1.04 (0.73-1.49)	4/49 (8.2)		0.86 (0.30-2.48)
≥2	32/73 (43.8)	3.07 (1.67-5.65)	12/67 (17.9)	2.43 (1.26-4.67)	31/37 (83.8)	2.01 (1.32-3.08)	6/34 (17.6)		1.89 (0.74-4.83)
Acute postoperative pain (BPI ≥4 at 3 d)									
No	1623/7081 (22.9)	1.0 [Reference]	505/5314 (9.5)	1.0 [Reference]	2762/3649 (75.7)	1.0 [Reference]	339/3523 (9.6)		1.0 [Reference]
Yes	241/890 (27.1)	1.41 (1.06-1.88)	42/337 (12.5)	1.28 (0.91-1.80)	160/196 (81.6)	1.33 (1.11-1.59)	19/188 (10.1)		0.97 (0.58-1.60)
Any postoperative complications^f^									
No	1350/5543 (24.4)	1.0 [Reference]	597/6844 (8.7)	1.0 [Reference]	3483/4648 (74.9)	1.0 [Reference]	416/4531 (9.2)		1.0 [Reference]
Yes	113/352 (32.1)	1.45 (1.18-1.79)	92/860 (10.7)	1.24 (0.97-1.58)	420/518 (81.1)	1.13 (1.00-1.27)	54/499 (10.8)		1.00 (0.73-1.38)

Abbreviations: AD8, 8-Item Informant Interview to Differentiate Aging and Dementia; ASA, American Society of Anesthesiologists; BPI, Brief Pain Inventory; GAD-7, Generalized Anxiety Disorder 7-item; ICU, intensive care unit; OR, odds ratio; PHQ-9, Patient Health Questionnaire 9; PSQI, Pittsburgh Sleep Quality Index; TRT, 3-Word Recall Test.

^a^ The ORs and 95% CIs were derived from generalized liner mixed-effect models, adjusted for age, sex, ethnicity, educational attainment, occupation, body mass index, status of smoking and drinking, and site and type of surgery.

^b^ The ORs and 95% CIs were derived from logistic regression models, adjusted for age, sex, ethnicity, educational attainment, occupation, body mass index, status of smoking and drinking, and site and type of surgery.

^c^ Pain duration of more than 1 month.

^d^ Oxygen saturation less than 90% during the surgery.

^e^ Atrial pressure less than 60% of baseline level during the surgery.

^f^ Included pulmonary complication, major adverse cardiac event, acute kidney injury, and infection.

**Table 3.** Associations of Studied Factors With the Occurrence or Aggressively Deteriorative Trajectory of Subjective Cognitive or Short-Term Memory Impairment Following Cardiac Surgery

Variable	Postoperative AD8 abnormality (AD8 ≥2, n = 678)		Aggressively deteriorative trajectory of AD8 scores (n = 635)		Postoperative TRT abnormality (TRT <3, n = 454)		Aggressively deteriorative trajectory of TRT scores (n = 432)	
	No./total No. (%) of cases	OR (95% CI)^a^	No./total No. (%) of cases	OR (95% CI)^b^	No./total No. (%) of cases	OR (95% CI)^a^	No./total No. (%) of cases	OR (95% CI)^b^
Preoperative comorbidities								
Psychiatric disorder								
No	212/672 (31.6)	1.0 [Reference]	53/630 (8.4)	1.0 [Reference]	395/447 (88.4)	1.0 [Reference]	134/425 (31.5)	1.0 [Reference]
Yes	1/6 (16.7)	0.78 (0.12-5.21)	1/5 (20.0)	2.20 (0.22-22.19)	7/7 (100)	0.71 (0.27-1.86)	2/7 (28.6)	0.97 (0.17-5.66)
Diabetes								
No	200/631 (31.7)	1.0 [Reference]	52/593 (8.8)	1.0 [Reference]	374/425 (88.0)	1.0 [Reference]	122/404 (30.2)	1.0 [Reference]
Yes	13/47 (27.7)	0.71 (0.35-1.46)	2/42 (4.8)	0.54 (0.12-2.38)	28/29 (96.6)	1.37 (0.79-2.36)	14/28 (50.0)	1.64 (0.68-3.92)
Hepatic disease								
No	182/593 (30.7)	1.0 [Reference]	45/555 (8.1)	1.0 [Reference]	352/396 (88.9)	1.0 [Reference]	117/376 (31.1)	1.0 [Reference]
Yes	31/85 (36.5)	1.23 (0.76-1.98)	9/80 (11.2)	1.51 (0.68-3.35)	50/58 (86.2)	1.10 (0.75-1.62)	19/56 (33.9)	1.33 (0.68-2.62)
Peptic ulcer								
No	198/636 (31.1)	1.0 [Reference]	48/595 (8.1)	1.0 [Reference]	380/430 (88.4)	1.0 [Reference]	129/408 (31.6)	1.0 [Reference]
Yes	15/42 (35.7)	1.70 (0.89-3.23)	6/40 (15.0)	2.75 (1.02-7.43)	22/24 (91.7)	0.72 (0.41-1.27)	7/24 (29.2)	0.78 (0.28-2.18)
Solid tumor								
No	213/676 (31.5)	1.0 [Reference]	54/634 (8.5)	1.0 [Reference]	401/452 (88.7)	1.0 [Reference]	136/431 (31.6)	1.0 [Reference]
Yes	0/2 (0)	0.00 (0.00-Inf)	0/1 (0)	0.00 (0.00-Inf)	1/2 (50.0)	0.74 (0.08-6.60)	0/1 (0)	0.00 (0.00-Inf)
Chronic pain^c^								
No	157/531 (29.6)	1.0 [Reference]	36/500 (7.2)	1.0 [Reference]	302/344 (87.8)	1.0 [Reference]	106/326 (32.5)	1.0 [Reference]
Yes	43/117 (36.8)	1.48 (0.96-2.26)	13/105 (12.4)	1.73 (0.86-3.50)	78/88 (88.6)	0.99 (0.71-1.37)	26/84 (31.0)	1.00 (0.57-1.78)
Pain in the week before surgery								
No	150/553 (27.1)	1.0 [Reference]	38/512 (7.4)	1.0 [Reference]	318/365 (87.1)	1.0 [Reference]	112/344 (32.6)	1.0 [Reference]
Yes	50/95 (52.6)	2.57 (1.72-3.85)	11/93 (11.8)	1.60 (0.77-3.34)	62/67 (92.5)	0.81 (0.56-1.15)	20/66 (30.3)	0.80 (0.42-1.49)
Psychological condition								
Anxiety (GAD-7 score)								
<5	190/639 (29.7)	1.0 [Reference]	48/597 (8.0)	1.0 [Reference]	378/426 (88.7)	1.0 [Reference]	127/405 (31.4)	1.0 [Reference]
≥5	23/39 (59.0)	2.43 (1.33-4.46)	6/38 (15.8)	1.63 (0.61-4.38)	24/28 (85.7)	1.49 (0.87-2.55)	9/27 (33.3)	1.54 (0.60-3.99)
Depression (PHQ-9 score)								
<5	181/611 (29.6)	1.0 [Reference]	45/575 (7.8)	1.0 [Reference]	357/399 (89.5)	1.0 [Reference]	122/380 (32.1)	1.0 [Reference]
≥5	32/67 (47.8)	2.39 (1.49-3.84)	9/60 (15.0)	1.95 (0.87-4.39)	45/55 (81.8)	0.88 (0.59-1.29)	14/52 (26.9)	0.97 (0.48-1.98)
Sleep disturbance (PSQI score)								
0-5	89/305 (29.2)	1.0 [Reference]	2/9 (22.2)	1.0 [Reference]	176/201 (87.6)	1.0 [Reference]	2/7 (28.6)	1.0 [Reference]
6-10	78/275 (28.4)	1.09 (0.77-1.55)	20/282 (7.1)	1.56 (0.84-2.90)	164/187 (87.7)	1.09 (0.83-1.44)	58/187 (31.0)	0.94 (0.57-1.54)
11-15	36/87 (41.4)	1.02 (0.62-1.69)	4/82 (4.9)	0.60 (0.19-1.87)	55/59 (93.2)	1.54 (1.02-2.32)	22/57 (38.6)	1.24 (0.62-2.50)
≥16	10/11 (90.9)	8.42 (3.03-23.37)	28/262 (10.7)	4.23 (0.75-23.96)	7/7 (100)	0.99 (0.39-2.54)	54/181 (29.8)	1.03 (0.18-5.86)
Anesthesia- or surgery-related factors								
Type of surgery								
Endoscopic surgery	8/26 (30.8)	1.0 [Reference]	2/20 (10.0)	1.0 [Reference]	16/19 (84.2)	1.0 [Reference]	6/17 (35.3)	1.0 [Reference]
Open surgery	205/652 (31.4)	0.41 (0.18-0.94)	52/615 (8.5)	0.60 (0.12-2.95)	386/435 (88.7)	0.59 (0.29-1.19)	130/415 (31.3)	0.58 (0.18-1.92)
Anesthesia duration, h								
≤6	164/533 (30.8)	1.0 [Reference]	43/503 (8.6)	1.0 [Reference]	319/364 (87.6)	1.0 [Reference]	110/348 (31.6)	1.0 [Reference]
>6	47/137 (34.3)	1.09 (0.73-1.63)	11/128 (8.6)	0.99 (0.48-2.07)	78/84 (92.9)	1.05 (0.75-1.48)	25/81 (30.9)	0.94 (0.52-1.70)
ASA grade								
<3	6/19 (31.6)	1.0 [Reference]	3/15 (20.0)	1.0 [Reference]	11/12 (91.7)	1.0 [Reference]	1/11 (9.1)	1.0 [Reference]
≥3	207/659 (31.4)	0.70 (0.27-1.84)	51/620 (8.2)	0.35 (0.09-1.38)	391/442 (88.5)	0.88 (0.38-2.05)	135/421 (32.1)	3.91 (0.46-32.96)
Type of general anesthesia maintenance								
Total intravenous anesthesia	16/32 (50.0)	1.0 [Reference]	5/32 (15.6)	1.0 [Reference]	18/20 (90.0)	1.0 [Reference]	6/20 (30.0)	1.0 [Reference]
Combined intravenous and inhalation anesthesia	192/638 (30.1)	0.68 (0.35-1.32)	49/595 (8.2)	0.43 (0.15-1.21)	378/428 (88.3)	1.04 (0.57-1.90)	128/406 (31.5)	1.64 (0.55-4.87)
Inhalation anesthesia	5/8 (62.5)	0.71 (0.16-3.07)	0/8 (0)	0.00 (0.00-Inf)	6/6 (100)	1.09 (0.33-3.59)	2/6 (33.3)	1.79 (0.23-13.94)
Combined with nerve block								
No	208/645 (32.2)	1.0 [Reference]	53/607 (8.7)	1.0 [Reference]	383/430 (89.1)	1.0 [Reference]	128/412 (31.1)	1.0 [Reference]
Yes	5/33 (15.2)	0.63 (0.23-1.69)	1/28 (3.6)	0.43 (0.05-3.36)	19/24 (79.2)	1.07 (0.57-2.01)	8/20 (40.0)	1.58 (0.56-4.48)
Intraoperative blood transfusion								
No	24/97 (24.7)	1.0 [Reference]	4/92 (4.4)	1.0 [Reference]	59/68 (86.8)	1.0 [Reference]	19/67 (28.4)	1.0 [Reference]
Yes	189/565 (33.4)	1.25 (0.77-2.04)	50/543 (9.2)	2.17 (0.75-6.29)	337/372 (90.6)	1.25 (0.87-1.79)	117/365 (32.0)	1.35 (0.70-2.59)
Severe hypoxia^d^								
No	158/517 (30.6)	1.0 [Reference]	36/487 (7.4)	1.0 [Reference]	309/350 (88.3)	1.0 [Reference]	107/335 (31.9)	1.0 [Reference]
Yes	53/153 (34.6)	1.39 (0.95-2.03)	18/144 (12.5)	1.99 (1.06-3.75)	88/98 (89.8)	1.10 (0.80-1.51)	28/94 (29.8)	1.24 (0.71-2.18)
Severe hypotension^e^								
No	26/103 (25.2)	1.0 [Reference]	6/97 (6.2)	1.0 [Reference]	64/75 (85.3)	1.0 [Reference]	22/72 (30.6)	1.0 [Reference]
Yes	185/567 (32.6)	1.56 (0.95-2.55)	48/534 (9.0)	1.61 (0.65-4.01)	333/373 (89.3)	1.18 (0.83-1.68)	113/357 (31.6)	1.20 (0.65-2.23)
Postsurgical events								
Patient-controlled analgesia								
No	209/664 (31.5)	1.0 [Reference]	54/622 (8.7)	1.0 [Reference]	390/441 (88.4)	1.0 [Reference]	131/419 (31.3)	1.0 [Reference]
Yes	4/14 (28.6)	1.23 (0.38-3.92)	0/13 (0)	0.00 (0.00-Inf)	12/13 (92.3)	0.92 (0.42-2.04)	5/13 (38.5)	1.20 (0.32-4.52)
Admission to ICU								
No	5/16 (31.2)	1.0 [Reference]	1/14 (7.1)	1.0 [Reference]	11/12 (91.7)	1.0 [Reference]	1/11 (9.1)	1.0 [Reference]
Yes	208/646 (32.2)	1.02 (0.36-2.90)	53/621 (8.5)	1.59 (0.19-13.13)	385/428 (90.0)	1.09 (0.50-2.37)	135/421 (32.1)	3.29 (0.39-27.64)
Length of ICU stay, d								
0	7/27 (25.93)	1.0 [Reference]	1/21 (4.8)	1.0 [Reference]	19/20 (95.0)	1.0 [Reference]	4/17 (23.5)	1.0 [Reference]
1	31/117 (26.5)	0.73 (0.29-1.89)	6/113 (5.3)	1.22 (0.13-11.20)	73/87 (83.9)	0.67 (0.33-1.34)	23/85 (27.1)	1.18 (0.32-4.37)
≥2	175/518 (33.8)	0.90 (0.38-2.13)	47/501 (9.4)	2.28 (0.29-18.16)	304/333 (91.3)	0.89 (0.46-1.71)	109/330 (33.0)	1.40 (0.41-4.71)
Acute postoperative pain (BPI ≥4 at 3 d)								
No	162/485 (33.4)	1.0 [Reference]	43/461 (9.3)	1.0 [Reference]	291/327 (89.0)	1.0 [Reference]	99/313 (31.6)	1.0 [Reference]
Yes	14/35 (40.0)	0.94 (0.47-1.90)	2/35 (5.7)	0.48 (0.11-2.17)	21/25 (84.0)	0.71 (0.40-1.26)	3/25 (12.0)	0.29 (0.07-1.12)
Any postoperative complications^f^								
No	74/256 (28.9)	1.0 [Reference]	20/243 (8.2)	1.0 [Reference]	157/179 (87.7)	1.0 [Reference]	47/173 (27.2)	1.0 [Reference]
Yes	139/406 (34.2)	1.29 (0.92-1.81)	34/392 (8.7)	1.15 (0.64-2.10)	239/261 (91.6)	1.17 (0.89-1.52)	89/259 (34.4)	1.33 (0.83-2.13)

Abbreviations: AD8, 8-Item Informant Interview to Differentiate Aging and Dementia; ASA, American Society of Anesthesiologists; BPI, Brief Pain Inventory; GAD-7, Generalized Anxiety Disorder 7-item; ICU, intensive care unit; OR, odds ratio; PHQ-9, Patient Health Questionnaire 9; PSQI, Pittsburgh Sleep Quality Index; TRT, 3-Word Recall Test.

^a^ The ORs and 95% CIs were derived from generalized liner mixed-effect models, adjusted for age, sex, ethnicity, educational attainment, occupation, body mass index, status of smoking and drinking, and site and type of surgery.

^b^ The ORs and 95% CIs were derived from logistic regression models, adjusted for age, sex, ethnicity, educational attainment, occupation, body mass index, status of smoking and drinking, and site and type of surgery.

^c^ Pain duration of more than 1 month.

^d^ Oxygen saturation less than 90% during the surgery.

^e^ Atrial pressure less than 60% of baseline level during the surgery.

^f^ Included pulmonary complication, major adverse cardiac event, acute kidney injury, and infection.

Estimates from the mutually adjusted models were slightly attenuated but remained largely similar (eTables 3 and 4 in Supplement 1). To facilitate comparisons with other studies, we reported ORs for sociodemographic and lifestyle factors (eTables 5 and 6 in Supplement 1). In addition, subgroup analyses suggested that, except for few studied factors (eg, psychiatric disorders and patient-controlled analgesia), most observed associations could not be modified by age or sex (eTables 7 and 8 in Supplement 1). The sensitivity analysis that used individuals clustered in the consistently low-risk trajectory as the reference group revealed results similar to those of the main analyses (eTable 9 in Supplement 1).

## Discussion

Using the CSAC database, we demonstrated the existence of subjective cognitive and short-term memory impairment within 12 months after cardiac and noncardiac surgery in the middle-aged Chinese population. More specifically, despite a generally higher score level among individuals undergoing cardiac surgery than those undergoing noncardiac surgery, the changing patterns of studied measurements were largely similar between those 2 populations. The incidence of AD8 abnormality notably increased and reached 17.1% at 6 months after the index noncardiac surgery and cardiac surgery, whereas the TRT abnormality illustrated a U-shaped trajectory with most pronounced estimates both immediately (7 days; 38.9% after noncardiac surgery and 57.7% after cardiac surgery) and at 12 months (49.0% after noncardiac surgery and 58.9% after cardiac surgery). Moreover, our risk factor analyses revealed the importance of pain in the week before surgery, some preoperative psychological conditions (eg, sleep disturbance and depressive symptoms), anesthesia- and surgery-related factors (eg, type of surgery and >3 hours of general anesthesia duration), and postsurgical events (eg, days of ICU stay and patient-controlled analgesia) on the occurrence of subjective cognitive and short-term memory impairment and/or the aggressively deteriorative trajectories of those cognitive measurements. Such findings imply that interventions targeting preoperative psychological adversities (eg, poor sleep quality and depressive symptoms), as well as optimized perioperative management (eg, pain control), might be effective approaches for postoperative cognitive impairment prevention.

Without comparable data retrieved from studies focusing on postoperative cognitive impairment among the middle-aged population in China, our results gained support from previous literature, including a study that recruited 580 middle-aged (40-60 years) patients from 5 European countries.^27^ Using a battery of neuropsychological tests as evaluation scales, this study found that the incidence of cognitive dysfunction was 19.2% at 7 days and 6.2% at 3 months after major abdominal or orthopedic surgery. Studies using screening scales generally obtained milder cases and thereby reported higher cognitive dysfunction rates than those using neuropsychological tests. In a comparative study, both the AD8 and MoCA were ranked top for recognizing mild cognitive impairment and dementia in elderly individuals, showing higher sensitivity and specificity than other screeners, such as Mini-Cog and Mini-Mental State Examination.^28^ Studies using the MoCA reported an incidence of 16.9% to 24.0% for postoperative cognitive decline within 3 months after surgery,^29,30^ which was higher but basically comparable with the estimates we obtained for subjective cognitive impairment indexed by AD8 abnormality among middle-aged Chinese patients. Similarly, in line with our findings, previous reports of small clinical studies noted a substantial proportion of patients with short-term memory impairment (41.0%-45.5%) after general anesthesia.^31,32^

Changes of postoperative cognitive function over time have been less studied primarily because of the lack of longitudinally collected data. In the present study, using a data-driven approach, we clustered patients according to their changing patterns of cognitive function measured at multiple follow-ups within the first year after surgery. Such analyses led to the identification of 3 groups of patients with distinct postoperative cognitive trajectories, labeled consistently low risk, recovering, and aggressively deteriorative. The existence of those 3 trajectories was commonly observed in both cardiac and noncardiac surgery cohorts and has been hypothesized in a previous review.^33^ We accordingly placed a particular focus on patients with an aggressively deteriorative trajectory because it presents a group with the highest possibility of benefiting from future early intervention.

Among the risk factors associated with both the occurrence of subjective cognitive and short-term memory impairment and their aggressively deteriorative trajectories after surgery, preoperative modifiable factors have great clinical importance. Accumulating evidence has indicated an association between preoperative psychological factors and altered cognitive function after surgery. For instance, an observational study of 148 patients undergoing cardiac surgery identified preoperative depressive symptoms as being associated with cognitive decline 1 month after surgery.^34^ Furthermore, in a randomized clinical trial involving 139 elderly patients undergoing hip arthroplasty, a protective effect of exogenous melatonin supplementation, as an intervention for sleep quality improvement among surgery patients, an early postoperative cognitive decline (at days 1, 3, 5, and 7) was demonstrated.^35^ Explanations for the observed links might include hippocampal atrophy, alterations in glucocorticoid secretion, and deposition of β-amyloid plaques that occurred during the pathogenesis of depression,^36^ as well as activated neuroinflammation and oxidative stress, impaired function of the blood-brain barrier and glymphatic pathway, and hippocampal pathology that was induced by sleep deprivation.^37^ Moreover, pain was reported to aggravate cognitive dysfunction mediated by changes in neurotransmitters and inflammatory factors.^38^

We found that anesthesia- or surgery-related factors and postsurgical events, such as more than 3 hours of general anesthesia, an ICU stay of 2 days or longer, acute postoperative pain, and the occurrence of any postoperative complications, were associated with subjective cognitive and/or short-term memory impairment after noncardiac surgery but not after cardiac surgery; these findings were somewhat different from previous reports that highlighted those factors mainly for cardiac surgery patients.^5,39,40^ However, the participants of those studies were older (mean or median age >60 years) and received coronary artery bypass grafting operations, whereas our cardiac surgery cohort included middle-aged participants who predominantly underwent valve replacements; thus the inconsistent results between our and prior investigations do not necessarily invalidate each other.

### Strengths and Limitations

The main strengths of our study include the large sample from the CSAC, which thus far has recruited more than 10 000 participants from multiple medical centers in China. In addition, the stringent data quality control processes together with timely updates of data from well-established electronic medical systems provide reliable and enriched data for risk factor identification, as well as enable the consideration of a wide range of covariates during analyses. Second, the measurements of postoperative cognitive function were obtained prospectively at multiple follow-up points, offering an opportunity for studying prolonged cognitive alterations after surgery. Third, the application of trajectory analysis successfully identified a group of patients with aggressively deteriorative cognitive function, and further risk factor analyses focusing on this group have the potential to detect factors that can effectively impede the cognitive decline among surgery patients.

There were also some notable limitations to the study. First, to increase the feasibility of cognitive function measured at multiple time points, which needs to be doable through both face-to-face interview (at baseline) and telephone or web-based follow-ups, we applied the AD8 scale as the main evaluation tool. Although widely used as a screening tool in other large-scale cohorts^22,23^ and achieving satisfactory consistencies with clinical assessment and neuropsychological tests in validation studies,^20,41,42^ the AD8 cannot be considered a diagnostic tool for cognitive dysfunction. The additional use of TRT might, to some extent, offer supplemental data, particularly for memory impairment.^25,28^ Nevertheless, future studies with more precise assessment approaches are warranted to verify our findings. Second, although we found that the presence of neither severe hypoxia nor severe hypotension was associated with subjective cognitive impairment, this result must be interpreted with caution because the duration of hypoxia and hypotension might matter more for cognitive outcomes. Third, despite the high response rate and follow-up rates, the participants were recruited from only 2 medical centers in Chengdu. The generalizability of our findings to the rest of China or other populations needs further evaluation.

## Conclusions

In this cohort study of middle-aged Chinese patients, we demonstrated the existence of subjective cognitive and short-term memory impairment within 12 months after both cardiac and noncardiac surgery. Additionally, preoperative pain and psychological conditions as well as longer duration of general anesthesia and ICU stay were identified as important risk factors associated with the presence and aggressively deteriorative trajectory of subjective cognitive impairment after surgery, highlighting the potential of preoperative psychological intervention and optimized perioperative management for postoperative cognitive impairment prevention.

## Notes

Supplement 1. **eMethods.** Supplementary Methods **eFigure 1.** Quality Checks to Ensure the Data Accuracy in the CSAC Database **eFigure 2.** Study Design **eFigure 3.** Mean Scores of the 8-Item Interview to Differentiate Aging and Dementia (AD-8) and Three-Word Recall Test (TRT) at Different Follow-Up Time Among Participants Receiving Noncardiac and Cardiac Surgery **eTable 1.** The Alzheimer’s Disease 8-Item Interview (AD-8) to Differentiate Aging and Dementia (AD-8) **eTable 2.** Measurement and Data Source of Potential Risk Factors of Interest **eTable 3.** Multivariable Analyses on the Risk Factors Associated With the Occurrence of the Aggressively Deteriorative Trajectory of Subjective Cognitive (Measured by 8-Item Informant Interview to Differentiate Aging and Dementia [AD-8] Score) or Short-Term Memory (Measured by Three-Word Recall Test [TRT] Score) Impairment Following Noncardiac Surgery **eTable 4.** Multivariable Analyses on the Risk Factors Associated With the Occurrence or the Aggressively Deteriorative Trajectory of Subjective Cognitive (Measured by 8-Item Informant Interview to Differentiate Aging and Dementia [AD-8] Score) or Short-Term Memory (Measured by Three-Word Recall Test [TRT] Score) Impairment Following Cardiac Surgery **eTable 5.** Association of Social Demographic/Lifestyle Factors With the Risk of the Occurrence or the Aggressively Deteriorative Trajectory of Subjective Cognitive (Measured by 8-Item Informant Interview to Differentiate Aging and Dementia [AD-8] Score) or Short-Term Memory (Measured by Three-Word Recall Test [TRT] Score) Impairment Following Noncardiac Surgery **eTable 6.** Association of Social Demographic/Lifestyle Factors With the Risk of the Occurrence or the Aggressively Deteriorative Trajectory of Subjective Cognitive (Measured by 8-Item Informant Interview to Differentiate Aging and Dementia [AD-8] Score) or Short-Term Memory (Measured by Three-Word Recall Test [TRT] Score) Impairment Following Cardiac Surgery **eTable 7.** Subgroup Analysis of Factors Associated With 8-Item Informant Interview to Differentiate Aging and Dementia [AD-8] Abnormality Among Participants Receiving Noncardiac Surgery **eTable 8.** Subgroup Analysis of Factors Associated With Short-Term Memory (Measured by Three-Word Recall Test [TRT] Score) Impairment Among Participants Receiving Noncardiac Surgery **eTable 9.** Sensitivity Analysis of Factors Associated With Aggressively Deteriorative Trajectory of 8-Item Informant Interview to Differentiate Aging and Dementia [AD-8] Scores and Three-Word Recall Test [TRT] Scores by Using the ‘Consistently Low-Risk Trajectory’ as the Reference Group

Supplement 2.
Data Sharing Statement
