# Supplementary material for: Postsurgery Subjective Cognitive and Short-Term Memory Impairment Among Middle-Aged Chinese Patients
Source: JAMA Netw Open. 2023 Oct 10;6(10):e2336985. doi: 10.1001/jamanetworkopen.2023.36985 (PMC10565601; doi:10.1001/jamanetworkopen.2023.36985)
Supplement: Supplement 1. — eMethods. Supplementary Methods eFigure 1. Quality Checks to Ensure the Data Accuracy in the CSAC Database eFigure 2. Study Design eFigure 3. Mean Scores of the 8-Item Interview to Differentiate Aging and Dementia (AD-8) and Three-Word Recall Test (TRT) at Different Follow-Up Time Among Participants Receiving Noncardiac and Cardiac Surgery eTable 1. The Alzheimer’s Disease 8-Item Interview (AD-8) to Differentiate Aging and Dementia (AD-8) eTable 2. Measurement and Data Source of Potential Risk Factors of Interest eTable 3. Multivariable Analyses on the Risk Factors Associated With the Occurrence of the Aggressively Deteriorative Trajectory of Subjective Cognitive (Measured by 8-Item Informant Interview to Differentiate Aging and Dementia [AD-8] Score) or Short-Term Memory (Measured by Three-Word Recall Test [TRT] Score) Impairment Following Noncardiac Surgery eTable 4. Multivariable Analyses on the Risk Factors Associated With the Occurrence or the Aggressively Deteriorative Trajectory of Subjective Cognitive (Measured by 8-Item Informant Interview to Differentiate Aging and Dementia [AD-8] Score) or Short-Term Memory (Measured by Three-Word Recall Test [TRT] Score) Impairment Following Cardiac Surgery eTable 5. Association of Social Demographic/Lifestyle Factors With the Risk of the Occurrence or the Aggressively Deteriorative Trajectory of Subjective Cognitive (Measured by 8-Item Informant Interview to Differentiate Aging and Dementia [AD-8] Score) or Short-Term Memory (Measured by Three-Word Recall Test [TRT] Score) Impairment Following Noncardiac Surgery eTable 6. Association of Social Demographic/Lifestyle Factors With the Risk of the Occurrence or the Aggressively Deteriorative Trajectory of Subjective Cognitive (Measured by 8-Item Informant Interview to Differentiate Aging and Dementia [AD-8] Score) or Short-Term Memory (Measured by Three-Word Recall Test [TRT] Score) Impairment Following Cardiac Surgery eTable 7. Subgroup Analysis of Factors Associated With [file jamanetwopen-e2336985-s001.pdf]

Supplementary Online Content

Yang L, Chen W, Yang D, et al. Postsurgery subjective cognitive and short-term memory impairment among middle-aged Chinese patients. *JAMA Netw Open*. 2023;6(10):e2336985. doi:10.1001/jamanetworkopen.2023.36985

eMethods. Supplementary Methods
eFigure 1. Quality Checks to Ensure the Data Accuracy in the CSAC Database
eFigure 2. Study Design
eFigure 3. Mean Scores of the 8-Item Interview to Differentiate Aging and Dementia (AD-8) and Three-word Recall Test (TRT) at Different Follow-Up Time Among Participants Receiving Noncardiac and Cardiac Surgery
eTable 1. The 8-Item Interview to Differentiate Aging and Dementia (AD-8)
eTable 2. Measurement and Data Source of Potential Risk Factors of Interest
eTable 3. Multivariable Analyses on the Risk Factors Associated With the Occurrence of the Aggressively Deteriorative Trajectory of Subjective Cognitive (Measured by 8-Item Interview to Differentiate Aging and Dementia [AD-8] Score) or Short-Term Memory (Measured by Three-word Recall Test [TRT] Score) Impairment Following Noncardiac Surgery
eTable 4. Multivariable Analyses on the Risk Factors Associated With the Occurrence or the Aggressively Deteriorative Trajectory of Subjective Cognitive (Measured by 8-Item Interview to Differentiate Aging and Dementia [AD-8] Score) or Short-Term Memory (Measured by Three-Word Recall Test [TRT] Score) Impairment Following Cardiac Surgery
eTable 5. Association of Social Demographic/Lifestyle Factors With the Risk of the Occurrence or the Aggressively Deteriorative Trajectory of Subjective Cognitive (Measured by 8-Item Interview to Differentiate Aging and Dementia [AD-8] Score) or Short-Term Memory (Measured by Three-Word Recall Test [TRT] Score) Impairment Following Noncardiac Surgery
eTable 6. Association of Social Demographic/Lifestyle Factors With the Risk of the Occurrence or the Aggressively Deteriorative Trajectory of Subjective Cognitive (Measured by 8-Item Interview to Differentiate Aging and Dementia [AD-8] Score) or Short-Term Memory (Measured by Three-Word Recall Test [TRT] Score) Impairment Following Cardiac Surgery
eTable 7. Subgroup Analysis of Factors Associated With 8-Item Interview to Differentiate Aging and Dementia [AD-8] Abnormality Among Participants Receiving Noncardiac Surgery
eTable 8. Subgroup Analysis of Factors Associated With Short-Term Memory (Measured by Three-Word Recall Test [TRT] Score) Impairment Among Participants Receiving Noncardiac Surgery
eTable 9. Sensitivity Analysis of Factors Associated With Aggressively Deteriorative Trajectory of 8-Item Interview to Differentiate Aging and Dementia [AD-8] Scores and Three-Word Recall Test [TRT] Scores by Using the 'Consistently Low-Risk Trajectory' as the Reference Group

This supplementary material has been provided by the authors to give readers additional information about their work.

© 2023 Yang L et al. *JAMA Network Open.*

eMethods. Supplementary Methods

Details of the CSAC

In brief, baseline data on sociodemographic characteristics, lifestyle, medical history, and preoperative psychological condition were collected during the initial recruitment process (i.e., one day before the planned surgery) through face-to-face interviews conducted by well-trained data collectors. We used the newly developed Cohort Data Collection and Management System (CD-CMS) (Version 1.0, Build 2021SR0484324. ©West China Hospital, Sichuan, China), which implemented multistep quality checks to ensure data accuracy (Supplementary Figure 1). After the surgery, active follow-ups before the patients' discharge (i.e., at 1 and 3 days after surgery) were also conducted by data collectors via ward visits, while the follow ups after hospital discharge (i.e., 7 days and 1, 3, 6, and 12 months after the surgery) were performed by telephone or online questionnaires delivered through the official WeChat account that was linked to the CD-CMS. Through data linkages, we were able to derive documented data about the surgery and general anesthesia procedures (e.g., continuously measures of arterial pressure, and pulse oxygen saturation [SpO2]) from the Anesthesia Information Management System (AIMS, available for West China Hospital and West China Tianfu Hospital), as well as detailed information about indication diseases, medical examinations, and interventions received during hospitalization from the electronic medical record (EMR) system (available for all of the included medical centers). The initial efforts were placed on non-cardiac surgeries (e.g., abdominal, thoracic, and otorhinolaryngologic surgery); however, we started the inclusion of cardiac surgeries in West China Hospital in July 2021 using a modified questionnaire.

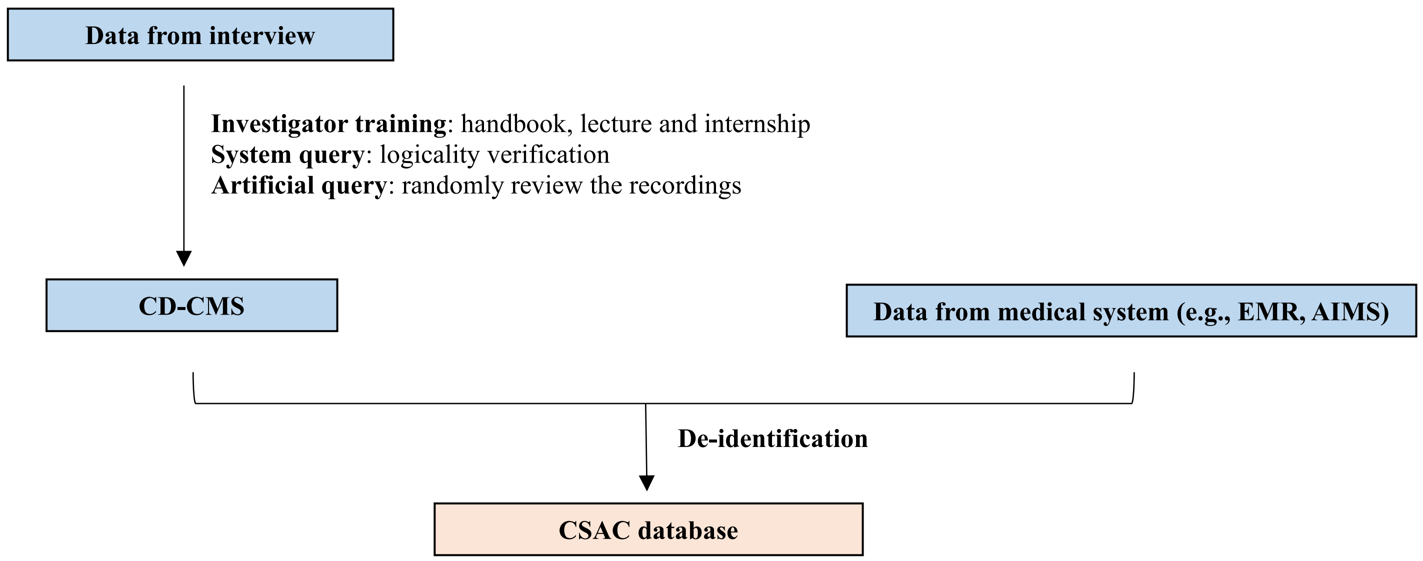

eFigure 1. Quality checks to ensure the data accuracy in the CSAC database
CD-CMS: Cohort Data Collection and Management System
CSAC: China Surgery and Anesthesia Cohort
EMR: Electronic Medical Record system
AIMS: Anesthesia Information Management System

© 2023 Yang L et al. *JAMA Network Open.*

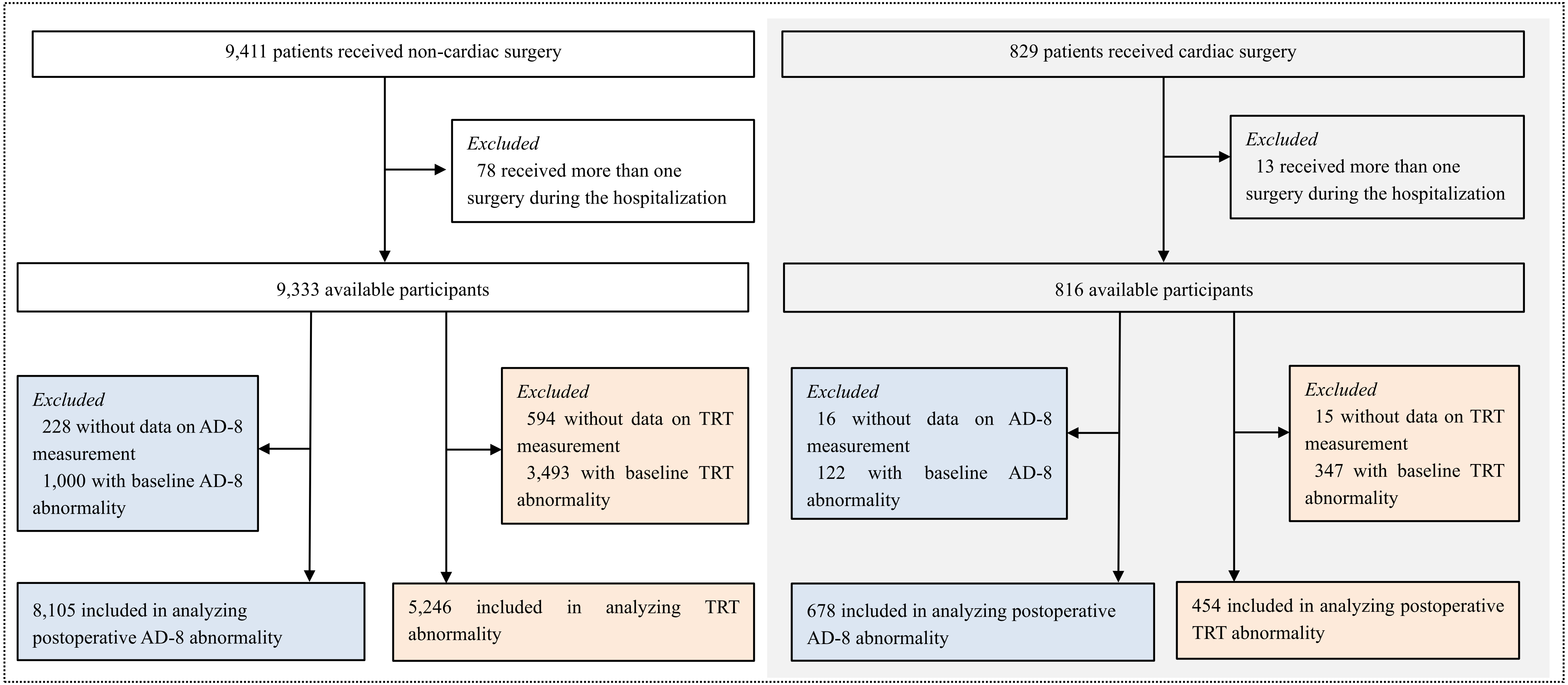

eFigure 2. Study design
a. AD-8 score ≥ 2 was considered as a cutoff point for the presence of subjective cognitive impairment
b. TRT score < 3 was considered as an indicator of short-term memory impairment.
AD-8: The 8-item Interview to Differentiate Aging and Dementia. TRT: Three-word recall test

© 2023 Yang L et al. *JAMA Network Open.*

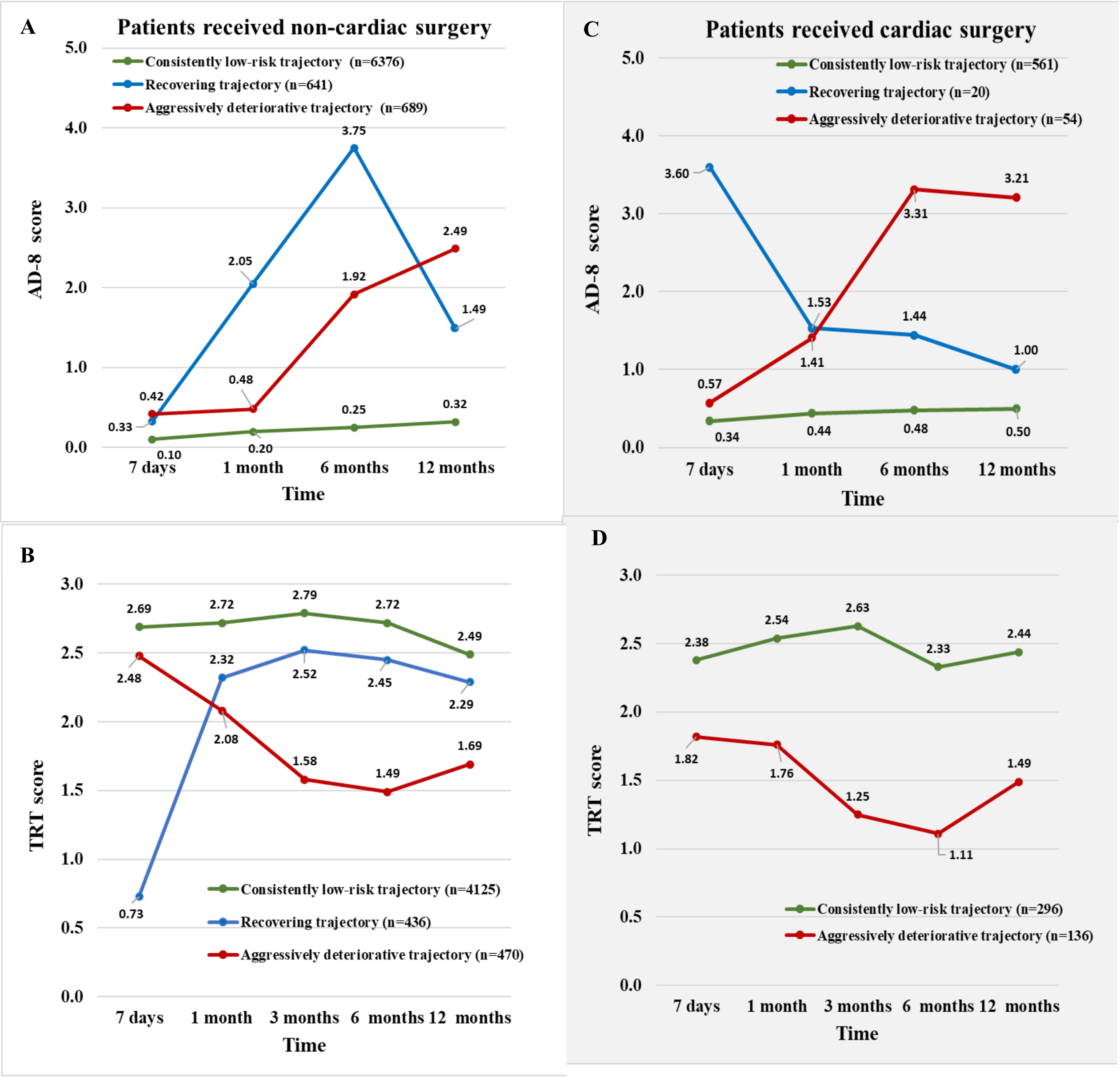

eFigure 3 Mean scores of the 8-item Interview to Differentiate Aging and Dementia (AD-8) and Three-word Recall Test (TRT), at different follow-up time among participants receiving noncardiac and cardiac surgery

AD-8 scores range from 0 to 8, with a higher score indicating potentially more severe subjective cognitive impairment. TRT scores range from 0 to 3, with a lower score indicating potentially more severe short-term memory impairment.

© 2023 Yang L et al. *JAMA Network Open.*

eTable 1 The Alzheimer's Disease 8-item interview (AD-8)

Remember, "Yes, a change" (1 point) indicates that there has been a change during a specified period caused by cognitive (thinking and memory) problems.	YES, A change	NO, No change	N/A, Don't know
1. Problems with judgment (e.g., problems making decisions, bad financial decisions, problems with thinking)			
2. Less interest in hobbies/activities			
3. Repeats the same things over and over (questions, stories, or statements)			
4. Trouble learning how to use a tool, appliance, or gadget (e.g., VCR, computer, microwave, remote control)			
5. Forgets correct month or year			
6. Trouble handling complicated financial affairs (e.g., balancing checkbook, income taxes, paying bills)			
7. Trouble remembering appointments			
8. Daily problems with thinking and/or memory			
TOTAL AD8 SCORE			

Adapted from Galvin JE et al, The AD-8, a brief informant interview to detect dementia, Neurology 2005:65:559-564. The AD-8 is a copyrighted instrument of the Alzheimer's Disease Research Center, Washington University, St. Louis, Missouri.

eTable 2 Measurement and data source of potential risk factors of interest

Potential risk factors of interest	Details of measurement	Data source
Preoperative comorbidities		
Psychiatric disorder	Yes / no	Preoperative interview
Diabetes	Yes / no	Preoperative interview
Hepatic disease	Yes / no	Preoperative interview
Peptic ulcer	Yes / no	Preoperative interview
Solid tumor	Yes / no	Preoperative interview
Chronic pain	Pain lasts more than 1 month (Yes / no)	Preoperative interview
Pain in the week before surgery	Pain in the week before surgery (Yes / no)	Preoperative interview
Psychological conditions		
Anxiety	Generalized Anxiety Disorder 7-item (GAD-7) [1]≥ 5	Preoperative interview
Depression	Patient Health Questionnaire-9 (PHQ-9) [2]≥ 5	Preoperative interview
Sleep disturbance	Pittsburgh sleep quality index (PSQI) [3]: mild: 1~5, moderate: 6~10, heavy: 11~15, and severe: 16~21	Preoperative interview
Anesthesia or Surgery-related		
Site of surgery	Head and neck, thorax, abdomen, limbs and others	Medical record
Type of surgery	Endoscopic or open surgery	Medical record
Anesthesia duration	≤ 3 hours, >3 hours in non-cardiac surgery; ≤ 6 hour, >6 hours in cardiac surgery	Medical record
American Society of Anesthesiologists (ASA) grade	I, II, III, IV, V	Medical record
Type of general anesthesia maintenance	Total intravenous anesthesia; Combined intravenous and inhalation anesthesia; Inhalation anesthesia	Medical record
Combined with nerve block	Yes / no	Medical record
Intraoperative blood transfusion	Yes / no	Medical record
Severe hypoxia	SpO_2 < 90% during the surgery (Yes / no)	Medical record
Severe hypotension	Atrial pressure < 60% of baseline level during the surgery (Yes / no)	Medical record
**Postsurgical events**		
Patient controlled analgesia	Yes / no	Medical record
Admission to ICU	Yes / no	Medical record
Length of ICU stay	days	Medical record
Acute postoperative pain	BPI < 4, ≥ 4 at 3 days after surgery	Postoperative interview
Any postoperative complications	Pulmonary complication, major adverse cardiac event, acute kidney injury, and infection (Yes / no)	Medical record

[1] Zhang, C., et al., Reliability, Validity, and Measurement Invariance of the General Anxiety Disorder Scale Among Chinese Medical University Students. Front Psychiatry, 2021. 12: p. 648755.
[2] Wang, W., et al., Reliability and validity of the Chinese version of the Patient Health Questionnaire (PHQ-9) in the general population. Gen Hosp Psychiatry, 2014. 36(5): p. 539-44.
[3] Tsai, P.S., et al., Psychometric evaluation of the Chinese version of the Pittsburgh Sleep Quality Index (CPSQI) in primary insomnia and control subjects. Qual Life Res, 2005. 14(8): p. 1943-52.

© 2023 Yang L et al. *JAMA Network Open.*

eTable 3 Multivariable analyses on the risk factors associated with the occurrence or the aggressively deteriorative trajectory of subjective cognitive (measured by 8-item Interview to Differentiate Aging and Dementia [AD-8] score) or short-term memory (measured by Three-word Recall Test [TRT] score) impairment following noncardiac surgery

Variable	Postoperative AD-8 abnormality (AD-8 ≥2, n=8,105)	Deteriorative trajectory of AD-8 scores (n=7,706)	Postoperative TRT abnormality (TRT<3, n=5,246)	Deteriorative trajectory of TRT scores (n=5,031)
	OR (95% CI) [a]	OR (95% CI) [b]	OR (95% CI) [a]	OR (95% CI) [b]
Comorbidity (yes vs no)				
Psychiatric disorder	1.36 (0.74-2.49)	1.44 (0.74-2.78)	1.22 (0.87-1.71)	1.56 (0.69-3.53)
Diabetes	0.84 (0.61-1.15)	1.20 (0.82-1.74)	1.06 (0.89-1.28)	0.96 (0.59-1.55)
Hepatic disease	1.11 (0.89-1.38)	1.18 (0.90-1.55)	1.07 (0.94-1.20)	0.94 (0.67-1.33)
Peptic ulcer	1.35 (0.94-1.95)	1.28 (0.84-1.96)	1.41 (1.14-1.73)	1.62 (0.96-2.74)
Solid tumor	1.08 (0.73-1.59)	1.00 (0.63-1.60)	1.06 (0.85-1.32)	1.25 (0.71-2.21)
Chronic pain [c]	1.20 (0.99-1.46)	1.05 (0.83-1.32)	0.98 (0.88-1.09)	0.99 (0.74-1.33)
Pain in the week before surgery	1.44 (1.11-1.86)	1.44 (1.07-1.94)	0.96 (0.82-1.11)	0.65 (0.40-1.04)
Psychological condition				
Anxiety (GAD-7 score ≥ 5 vs <5)	1.20 (0.84-1.70)	1.01 (0.67-1.54)	0.95 (0.79-1.14)	1.35 (0.81-2.26)
Depression (PHQ-9 score ≥ 5 vs <5)	1.51 (1.13-2.02)	1.41 (1.01-1.96)	1.05 (0.90-1.23)	0.98 (0.64-1.52)
Sleep disturbance (PSQI score)				
0-5	Ref	Ref	Ref	Ref
6-10	1.69 (1.42-2.00)	1.45 (1.17-1.79)	1.20 (1.09-1.32)	1.25 (0.96-1.63)
11-15	2.34 (1.80-3.03)	1.63 (1.19-2.22)	1.27 (1.10-1.48)	1.73 (1.18-2.53)
≥ 16	2.69 (1.26-5.76)	2.81 (1.30-6.08)	1.39 (0.90-2.16)	0.00 (0.00-Inf)
Anesthesia or Surgery-related				
Site of surgery				
Head and neck	Ref	Ref	Ref	Ref
Thorax	0.66 (0.46-0.95)	0.99 (0.62-1.57)	0.88 (0.72-1.07)	0.66 (0.37-1.15)
Abdomen	0.74 (0.57-0.98)	1.18 (0.83-1.68)	0.98 (0.84-1.13)	0.86 (0.57-1.30)
Limbs and surface	0.83 (0.62-1.12)	1.43 (1.00-2.05)	0.82 (0.70-0.97)	0.84 (0.53-1.31)
Other	0.76 (0.44-1.29)	0.94 (0.47-1.89)	1.09 (0.81-1.48)	0.47 (0.19-1.17)
Type of surgery (Open vs Endoscopic)	1.05 (0.87-1.27)	1.14 (0.90-1.44)	1.01 (0.90-1.12)	1.19 (0.89-1.59)
Anesthesia duration (> 3 hours vs ≤ 3 hours)	1.36 (1.12-1.65)	1.11 (0.88-1.41)	1.12 (1.01-1.25)	1.14 (0.85-1.52)
ASA grade (≥ 3 vs <3)	0.70 (0.53-0.94)	0.86 (0.61-1.21)	1.01 (0.86-1.18)	1.12 (0.76-1.66)
Type of general anesthesia maintenance				
Total intravenous anesthesia	Ref	Ref	Ref	Ref
Combined intravenous and inhalation anesthesia	0.85 (0.60-1.22)	0.79 (0.51-1.21)	1.02 (0.82-1.26)	0.62 (0.36-1.05)
Inhalation anesthesia	0.90 (0.40-2.01)	1.98 (0.88-4.45)	0.91 (0.59-1.42)	1.23 (0.44-3.47)
Nerve block (yes vs no)	1.07 (0.90-1.28)	1.05 (0.85-1.30)	1.00 (0.91-1.10)	1.23 (0.94-1.61)
Blood transfusion (yes vs no)	1.01 (0.67-1.53)	0.87 (0.53-1.44)	1.15 (0.91-1.46)	0.80 (0.44-1.47)
Severe hypoxia [d] (yes vs no)	0.66 (0.45-0.96)	0.75 (0.49-1.17)	1.15 (0.95-1.40)	1.39 (0.87-2.20)
Severe hypotension [e] (yes vs no)	0.82 (0.67-0.99)	0.92 (0.73-1.17)	1.03 (0.93-1.15)	1.18 (0.89-1.57)
**Postsurgical events**				
Patient-controlled analgesia (yes vs no)	1.08 (0.87-1.33)	1.15 (0.89-1.50)	1.05 (0.93-1.17)	1.02 (0.74-1.40)
Admission to ICU (yes vs no)	2.65 (0.52-13.42)	1.33 (0.16-11.37)	1.10 (0.42-2.93)	3.67 (0.55-24.57)

Continued on next page

© 2023 Yang L et al. *JAMA Network Open.*

Variables	Postoperative AD-8 abnormality (AD-8 ≥2, n=8,105)	Deteriorative trajectory of AD-8 scores (n=7,706)	Postoperative TRT abnormality (TRT<3, n=5,246)	Deteriorative trajectory of TRT scores (n=5,031)
	OR (95% CI) [a]	OR (95% CI) [b]	OR (95% CI) [a]	OR (95% CI) [b]
Length of ICU stay,d				
0	Ref	Ref	Ref	Ref
1	0.39 (0.07-2.27)	0.90 (0.09-9.03)	1.12 (0.39-3.22)	0.32 (0.04-2.84)
≥ 2	0.87 (0.14-5.23)	1.57 (0.16-15.93)	1.38 (0.47-4.11)	0.65 (0.07-5.90)
Acute postoperative pain (BPI ≥ 4 vs <4 at 3 days)	1.28 (0.94-1.76)	1.18 (0.81-1.73)	1.28 (1.07-1.55)	0.80 (0.47-1.38)
Any Postoperative complications [f] (yes vs no)	1.31 (1.02-1.67)	0.97 (0.72-1.31)	1.04 (0.90-1.19)	0.76 (0.51-1.13)

a OR and 95%CI were derived from generalized liner mixed-effect models, mutually adjusted for those studied factors, as well as age, sex, ethnicity, educational attainment, occupation, BMI, smoking, and drinking.
b OR and 95%CI were derived from logistic regression models, mutually adjusted for those studied factors, as well as age, sex, ethnicity, educational attainment, occupation, BMI, smoking, and drinking.
c Pain lasts more than 1 month
d SpO2 < 90% during the surgery;
e Atrial pressure < 60% of baseline level during the surgery;
f Included pulmonary complication, major adverse cardiac event, acute kidney injury, and infection.
Abbreviation: OR: Odds Ratio, CI: confidence interval, GAD-7: Generalized Anxiety Disorder 7-item, PHQ-9: Patient Health Questionnaire-9, PSQI: Pittsburgh sleep quality index; ASA: American Society of Anesthesiologists, ICU: Intensive care unit.

© 2023 Yang L et al. *JAMA Network Open.*

eTable 4 Multivariable analyses on the risk factors associated with the occurrence or the aggressively deteriorative trajectory of subjective cognitive (measured by 8-item Interview to Differentiate Aging and Dementia [AD-8] score) or short-term memory (measured by Three-word Recall Test [TRT] score) impairment following cardiac surgery

Variable	Postoperative AD-8 abnormality (AD-8 ≥ 2, n=678)	Aggressively deteriorative trajectory of AD-8 scores (n=635)	Postoperative TRT abnormality (TRT<3, n=454)	Aggressively Deteriorative trajectory of TRT scores (n=432)
	OR (95% CI) [a]	OR (95% CI) [b]	OR (95% CI) [a]	OR (95% CI) [b]
Comorbidity (yes vs no)				
Psychiatric disorder	0.00 (0.00-Inf)	0.00 (0.00-Inf)	0.41 (0.13-1.28)	0.32 (0.03-4.00)
Diabetes	1.02 (0.47-2.25)	1.48 (0.28-7.72)	1.43 (0.70-2.90)	2.82 (0.86-9.27)
Hepatic disease	1.31 (0.79-2.16)	1.79 (0.68-4.67)	0.96 (0.60-1.53)	1.33 (0.54-3.27)
Peptic ulcer	0.72 (0.32-1.59)	1.71 (0.42-6.96)	0.61 (0.30-1.21)	0.67 (0.18-2.49)
Solid tumor	0.00 (0.00-Inf)	-	0.00 (0.00-Inf)	-
Chronic pain [c]	1.01 (0.64-1.60)	1.31 (0.54-3.18)	1.11 (0.75-1.65)	0.98 (0.47-2.06)
Pain in the week before surgery	2.45 (1.59-3.80)	1.23 (0.47-3.26)	0.78 (0.50-1.22)	0.75 (0.33-1.73)
Psychological condition				
Anxiety (GAD-7 score ≥5 vs <5)	2.07 (1.06-4.05)	2.50 (0.67-9.29)	1.71 (0.85-3.46)	2.66 (0.67-10.52)
Depression (PHQ-9 score ≥ 5 vs <5)	1.23 (0.63-2.43)	1.22 (0.28-5.22)	0.87 (0.47-1.59)	0.97 (0.30-3.10)
Sleep disturbance (PSQI score)				
0-5	Ref	Ref	Ref	Ref
6-10	0.82 (0.55-1.22)	1.07 (0.48-2.37)	1.26 (0.88-1.81)	1.07 (0.55-2.08)
11-15	0.62 (0.33-1.15)	0.39 (0.09-1.66)	1.94 (1.10-3.43)	1.23 (0.47-3.25)
≥ 16	7.39 (2.28-23.92)	5.82 (0.54-63.03)	1.13 (0.29-4.32)	0.65 (0.05-8.45)
Anesthesia/Surgery-related				
Type of surgery (Open vs Endoscopic)	0.19 (0.08-0.45)	0.31 (0.05-1.97)	0.58 (0.23-1.43)	0.47 (0.08-2.71)
Anesthesia duration (>6 hours vs ≤ 6 hours)	0.87 (0.53-1.44)	0.77 (0.28-2.11)	0.78 (0.49-1.25)	0.79 (0.33-1.91)
ASA grade (≥ 3 vs <3)	0.61 (0.16-2.30)	0.27 (0.02-3.13)	0.54 (0.15-1.89)	2.27 (0.17-29.67)
Type of general anesthesia maintenance				
Total intravenous anesthesia	Ref	Ref	Ref	Ref
Combined intravenous and inhalation anesthesia	0.61 (0.31-1.22)	0.29 (0.08-0.99)	0.92 (0.44-1.92)	1.12 (0.31-4.11)
Inhalation anesthesia	0.87 (0.19-4.05)	0.00 (0.00-Inf)	1.34 (0.24-7.33)	2.10 (0.12-37.86)
Nerve block (yes vs no)	0.93 (0.28-3.12)	1.73 (0.19-15.98)	1.12 (0.40-3.09)	2.57 (0.42-15.90)
Blood transfusion (yes vs no)	0.52 (0.24-1.11)	0.53 (0.09-3.09)	1.28 (0.66-2.48)	0.89 (0.24-3.25)
Severe hypoxia [d] (yes vs no)	1.65 (1.07-2.55)	2.26 (0.97-5.25)	1.13 (0.75-1.68)	1.32 (0.64-2.74)
Severe hypotension [e] (yes vs no)	1.86 (0.95-3.63)	1.66 (0.44-6.31)	0.89 (0.54-1.47)	0.68 (0.28-1.65)
**Postsurgical events**				
Patient-controlled analgesia (yes vs no)	1.05 (0.28-3.93)	0.00 (0.00-Inf)	0.72 (0.25-2.09)	0.44 (0.05-3.53)
Admission to ICU (yes vs no)	10.43 (0.71-152.16)	10.20 (0.00-Inf)	2.63 (0.40-17.20)	1.03 (0.04-24.06)
Length of ICU stay, d				
0	Ref	Ref	Ref	Ref
1	0.39 (0.09-1.70)	-	0.27 (0.06-1.19)	0.89 (0.10-8.06)
≥ 2	0.31 (0.07-1.42)	-	0.30 (0.07-1.38)	0.81 (0.08-8.17)
Acute postoperative pain (BPI ≥ 4 vs < 4 at 3 days)	0.75 (0.36-1.55)	0.22 (0.03-2.00)	0.65 (0.35-1.24)	0.29 (0.07-1.21)
Any Postoperative complications [f] (yes vs no)	1.26 (0.85-1.86)	1.04 (0.48-2.26)	1.27 (0.88-1.83)	1.29 (0.64-2.57)

a OR and 95%CI were derived from generalized liner mixed-effect models, mutually adjusted for those studied factors, as well as age, sex, ethnicity, educational attainment, occupation, BMI, smoking, and drinking
b OR and 95%CI were derived from logistic regression models, mutually adjusted for those studied factors, as well as age, sex, ethnicity, educational attainment, occupation, BMI, smoking, and drinking
c Pain lasts more than 1 month
d SpO_2 < 90% during the surgery;
e Atrial pressure < 60% of baseline level during the surgery;
f Included pulmonary complication, major adverse cardiac event, acute kidney injury, and infection.

© 2023 Yang L et al. *JAMA Network Open.*

Abbreviation: OR: Odds Ratio, CI: confidence interval, GAD-7: Generalized Anxiety Disorder 7-item, PHQ-9: Patient Health Questionnaire-9, PSQI: Pittsburgh sleep quality index; ASA: American Society of Anesthesiologists, ICU: Intensive care unit.

© 2023 Yang L et al. *JAMA Network Open.*

eTable 5 Association of social demographic/lifestyle factors with risk of the occurrence or the aggressively deteriorative trajectory of subjective cognitive (measured by 8-item Interview to Differentiate Aging and Dementia [AD-8] score) or short-term memory (measured by Three-word Recall Test [TRT] score) impairment following non-cardiac surgery

Variables	Postoperative AD-8 abnormality (AD-8 ≥ 2, n=8,105)	Aggressively deteriorative trajectory of AD-8 scores (n=7,706)	Postoperative TRT abnormality (TRT<3, n=5,246)	Aggressively deteriorative trajectory of TRT scores (n=5,031)
	OR (95% CI) [a]	OR (95% CI) [b]	OR (95% CI) [a]	OR (95% CI) [b]
Time of measurement				
7 days	Ref	-	Ref	-
1 months	4.02 (3.21-5.03)	-	0.69 (0.62-0.77)	-
3 months	-	-	0.58 (0.52-0.65)	-
6 months	14.71 (11.76-18.40)	-	0.84 (0.75-0.95)	-
12 months	14.10 (11.20-17.75)	-	2.13 (1.89-2.41)	-
Social demographic data				
Age	1.02 (1.00-1.04)	1.02 (1.00-1.04)	1.03 (1.03-1.04)	1.05 (1.02-1.07)
Sex (female vs male)	1.40 (1.11-1.77)	1.55 (1.14-2.11)	0.98 (0.86-1.11)	1.06 (0.73-1.54)
Ethnicity (Others vs Han)	0.73 (0.31-1.70)	1.05 (0.42-2.59)	0.75 (0.48-1.17)	0.80 (0.23-2.74)
Education				
Middle school and lower	Ref	Ref	Ref	Ref
High school	0.72 (0.58-0.90)	0.77 (0.58-1.00)	0.66 (0.57-0.75)	0.47 (0.35-0.65)
Junior college	0.55 (0.43-0.70)	0.78 (0.58-1.05)	0.51 (0.44-0.58)	0.30 (0.21-0.43)
College and above	0.40 (0.31-0.52)	0.52 (0.38-0.73)	0.37 (0.32-0.43)	0.18 (0.12-0.28)
Occupation				
Blue collar	Ref	Ref	Ref	Ref
White collar	0.76 (0.55-1.06)	1.06 (0.71-1.60)	0.80 (0.66-0.97)	0.61 (0.38-0.98)
Self-employed	0.79 (0.56-1.09)	1.25 (0.83-1.88)	0.85 (0.69-1.05)	0.74 (0.46-1.20)
Retired	0.76 (0.55-1.04)	0.97 (0.65-1.44)	0.79 (0.65-0.97)	0.74 (0.47-1.17)
Others	0.83 (0.56-1.21)	0.93 (0.58-1.51)	0.98 (0.77-1.24)	0.85 (0.50-1.45)
Lifestyle factors				
BMI				
<18.5	Ref	Ref	Ref	Ref
18.5-25	0.96 (0.64-1.43)	0.98 (0.61-1.57)	1.15 (0.93-1.43)	1.94 (0.91-4.13)
26-30	1.06 (0.69-1.62)	0.90 (0.54-1.51)	1.34 (1.06-1.69)	2.02 (0.92-4.45)
≥ 30	0.90 (0.47-1.73)	0.75 (0.32-1.74)	1.04 (0.74-1.48)	1.47 (0.48-4.47)
Smoking (yes vs no)	0.90 (0.70-1.16)	1.11 (0.80-1.53)	1.08 (0.95-1.24)	1.19 (0.81-1.73)
Drinking (yes vs no)	0.86 (0.67-1.10)	0.82 (0.60-1.12)	0.86 (0.75-0.98)	1.01 (0.71-1.44)

[a] OR and 95%CI were derived from generalized liner mixed-effect models, mutually adjusted for all those studied factors
[b] OR and 95%CI were derived from logistic regression models, mutually adjusted for all those studied factors
OR: Odds Ratio, CI: confidence interval, BMI: body mass index.

© 2023 Yang L et al. *JAMA Network Open.*

eTable 6 Association of social demographic/lifestyle factors with risk of the occurrence or the aggressively deteriorative trajectory of subjective cognitive (measured by 8-item Interview to Differentiate Aging and Dementia [AD-8] score) or short-term memory (measured by Three-word Recall Test [TRT] score) impairment following cardiac surgery

Variables	Postoperative AD-8 abnormality (AD-8 ≥ 2, n=678)	Aggressively deteriorative trajectory of AD-8 scores (n=635)	Postoperative TRT abnormality (TRT<3, n=454)	Aggressively deteriorative trajectory of TRT scores (n=432)
	OR (95% CI) [a]	OR (95% CI) [b]	OR (95% CI) [a]	OR (95% CI) [b]
Time of measurement				
7 days	Ref	-	Ref	-
1 months	1.35 (0.86-2.11)	-	0.72 (0.50-1.03)	-
3 months	-	-	0.75 (0.52-1.07)	-
6 months	2.01 (1.29-3.13)	-	1.86 (1.26-2.75)	-
12 months	2.44 (1.52-3.93)	-	1.19 (0.77-1.83)	-
Social demographic data				
Age	0.99 (0.96-1.03)	0.98 (0.91-1.05)	1.07 (1.07-1.08)	1.11 (1.04-1.18)
Sex (female vs male)	1.37 (0.82-2.26)	1.83 (0.62-5.40)	1.26 (0.81-1.98)	1.21 (0.53-2.78)
Race (Others vs Han)	0.59 (0.16-2.14)	0.00 (0.00-Inf)	1.36 (0.57-3.25)	6.05 (1.16-31.59)
Education				
Middle school and lower	Ref	Ref	Ref	Ref
High school	0.98 (0.57-1.69)	0.80 (0.25-2.56)	0.78 (0.50-1.20)	0.56 (0.27-1.19)
Junior college	1.21 (0.64-2.31)	2.22 (0.67-7.36)	0.59 (0.34-1.02)	0.48 (0.16-1.39)
College and above	0.92 (0.40-2.11)	0.59 (0.10-3.49)	0.36 (0.19-0.69)	0.09 (0.01-0.57)
Occupation				
Blue collar	Ref	Ref	Ref	Ref
White collar	0.55 (0.28-1.08)	0.38 (0.09-1.66)	0.71 (0.40-1.28)	0.38 (0.13-1.10)
Self-employed	0.40 (0.20-0.81)	0.69 (0.19-2.52)	0.69 (0.40-1.21)	0.55 (0.21-1.42)
Retired	0.80 (0.42-1.50)	0.76 (0.21-2.76)	0.45 (0.25-0.78)	0.25 (0.09-0.72)
Others	1.12 (0.65-1.94)	1.35 (0.46-3.91)	0.82 (0.46-1.46)	0.83 (0.32-2.13)
Lifestyle factors				
BMI				
<18.5	Ref	Ref	Ref	Ref
18.5-25	0.76 (0.27-2.16)	0.16 (0.03-0.78)	1.44 (0.60-3.47)	2.02 (0.31-13.10)
26-30	0.63 (0.21-1.84)	0.10 (0.02-0.54)	1.51 (0.60-3.75)	1.50 (0.22-10.26)
≥ 30	0.56 (0.15-2.12)	0.19 (0.02-1.72)	1.30 (0.39-4.33)	3.51 (0.33-37.80)
Smoking (yes vs no)	0.83 (0.50-1.39)	0.84 (0.29-2.40)	1.55 (1.00-2.43)	1.79 (0.74-4.31)
Drinking (yes vs no)	1.09 (0.66-1.81)	1.89 (0.69-5.18)	0.51 (0.34-0.78)	0.33 (0.14-0.77)

[a] OR and 95%CI were derived from generalized liner mixed-effect models, mutually adjusted for all those studied factors
[b] OR and 95%CI were derived from logistic regression models, mutually adjusted for all those studied factors
OR: Odds Ratio, CI: confidence interval, BMI: body mass index.

© 2023 Yang L et al. *JAMA Network Open.*

eTable 7 Subgroup analysis of factors associated with 8-item Interview to Differentiate Aging and Dementia [AD-8] abnormality among participants receiving noncardiac surgery

Variables	By sex			By age		
	Male	Female	p for interaction	40-55 years	55-65 years	p for interaction
	OR (95% CI)	OR (95% CI)		OR (95% CI)	OR (95% CI)	
Comorbidity (yes vs no)						
Psychiatric disorder	0.28 (0.04-2.04)	2.35 (1.44-3.84)	0.0236	2.07 (1.08-3.98)	1.99 (0.97-4.09)	0.7485
Diabetes	0.75 (0.49-1.14)	0.97 (0.65-1.44)	0.5333	0.94 (0.58-1.50)	0.97 (0.71-1.31)	0.7701
Hepatic disease	1.08 (0.76-1.53)	1.17 (0.91-1.49)	0.7691	1.21 (0.92-1.57)	1.33 (1.00-1.78)	0.2824
Peptic ulcer	1.26 (0.74-2.17)	1.22 (0.78-1.90)	0.9696	0.92 (0.55-1.53)	1.70 (1.13-2.56)	0.0667
Solid tumor	1.54 (0.79-3.01)	0.98 (0.67-1.42)	0.3146	1.00 (0.61-1.64)	1.42 (0.94-2.13)	0.3436
Chronic pain [c]	1.43 (1.03-1.98)	1.52 (1.25-1.86)	0.2639	1.39 (1.09-1.76)	1.59 (1.24-2.03)	0.3732
Pain in the week before surgery	1.17 (0.72-1.89)	1.71 (1.31-2.24)	0.5862	2.18 (1.63-2.92)	0.88 (0.58-1.35)	0.0011
Psychological condition						
Anxiety (GAD-7 score ≥5 vs <5)	0.85 (0.41-1.79)	1.92 (1.44-2.56)	0.0889	1.67 (1.19-2.33)	2.66 (1.63-4.36)	0.1065
Depression (PHQ-9 score ≥5 vs <5)	1.57 (0.92-2.66)	2.45 (1.92-3.11)	0.0543	2.04 (1.54-2.71)	2.72 (1.94-3.80)	0.4852
Sleep disturbance (PSQI score)						
0-5	Ref	Ref		Ref	Ref	
6-10	1.87 (1.43-2.44)	1.60 (1.36-1.90)	0.8193	1.88 (1.54-2.30)	1.35 (1.09-1.67)	0.0698
11-15	2.79 (1.74-4.47)	2.36 (1.86-2.99)	0.7394	2.79 (2.06-3.78)	2.39 (1.77-3.23)	0.7001
≥16	1.73 (0.16-19.24)	3.87 (2.05-7.32)	0.5579	3.02 (1.10-8.27)	4.67 (2.10-10.37)	0.6471
Anesthesia/Surgery-related						
Site of surgery						
Head and neck	Ref	Ref	0.2485	Ref	Ref	
Thorax	1.27 (0.78-2.05)	0.82 (0.62-1.09)	0.0056	0.89 (0.64-1.23)	0.96 (0.66-1.39)	0.7193
Abdomen	1.85 (1.30-2.62)	0.87 (0.69-1.10)	0.4605	1.09 (0.85-1.41)	1.13 (0.84-1.52)	0.5114
Limbs and surface	0.72 (0.35-1.49)	0.99 (0.78-1.26)	0.2622	1.08 (0.82-1.43)	0.81 (0.55-1.19)	0.16
Others	1.02 (0.53-1.96)	0.54 (0.35-0.83)		0.58 (0.36-0.95)	0.62 (0.36-1.05)	0.8646
Type of surgery						
Open vs Endoscopic	1.42 (1.07-1.89)	1.12 (0.93-1.37)	0.3296	1.17 (0.95-1.45)	1.55 (1.22-1.96)	0.0172
Anesthesia duration						
> 3 hours vs ≤ 3 hours	1.73 (1.32-2.26)	1.29 (1.06-1.57)	0.0977	1.41 (1.14-1.75)	1.34 (1.07-1.69)	0.5051
ASA grade (⩾3 vs <3)	1.41 (0.96-2.05)	0.69 (0.49-0.96)	0.0248	0.94 (0.64-1.39)	0.99 (0.74-1.32)	0.4525
Type of general anesthesia maintenance						
Total intravenous anesthesia	Ref	Ref		Ref	Ref	
Combined intravenous and inhalation anesthesia	0.53 (0.29-0.96)	0.87 (0.62-1.23)	0.3187	0.85 (0.85-0.85)	0.77 (0.49-1.19)	0.972
Inhalation anesthesia	0.33 (0.09-1.22)	1.11 (0.49-2.49)	0.2059	0.55 (0.55-0.55)	0.83 (0.33-2.10)	0.8483
Nerve block (yes vs no)	1.20 (0.91-1.60)	1.05 (0.87-1.27)	0.3046	1.14 (0.92-1.40)	1.11 (0.89-1.37)	0.7832
Blood transfusion (yes vs no)	1.26 (0.72-2.21)	1.14 (0.73-1.79)	0.1641	1.10 (0.66-1.82)	1.73 (1.12-2.66)	0.3726
Severe hypoxia [a] (yes vs no)	1.49 (0.90-2.45)	0.57 (0.38-0.87)	0.0069	0.88 (0.55-1.42)	0.77 (0.51-1.16)	0.682
Severe hypotension [b] (yes vs no)	0.69 (0.50-0.95)	0.93 (0.76-1.14)	0.2429	0.94 (0.94-0.94)	0.72 (0.57-0.91)	0.3166

Continued on next page

© 2023 Yang L et al. *JAMA Network Open.*

Variables	By sex			By age		
	Male OR (95% CI)	Female OR (95% CI)	p for interaction	40-55 years OR (95% CI)	55-65 years OR (95% CI)	p for interaction
**Postsurgical events**						
Patient-controlled analgesia (yes vs no)	1.77 (1.31-2.40)	1.05 (0.84-1.30)	0.0073	1.30 (1.03-1.64)	1.37 (1.07-1.75)	0.1929
Admission to ICU (yes vs no)	1.89 (1.00-3.54)	1.78 (0.91-3.47)	0.6763	1.72 (0.93-3.17)	2.02 (1.07-3.80)	0.7436
Acute postoperative pain (BPI ⩾ 4 vs < 4 at 3 days)	1.20 (0.69-2.07)	1.65 (1.18-2.32)	0.1073	1.40 (0.94-2.08)	1.41 (0.92-2.15)	0.7094
Any Postoperative complications [c] (yes vs no)	1.72 (1.23-2.41)	1.16 (0.87-1.54)	0.1721	1.43 (1.06-1.92)	1.57 (1.19-2.08)	0.4543

[a] SpO2 < 90% during the surgery;
[b] Atrial pressure < 60% of baseline level during the surgery;
[c] Included pulmonary complication, major adverse cardiac event, acute kidney injury, and infection.
OR and 95%CI were derived from generalized liner mixed-effect models, adjusted for age, sex, ethnicity, educational attainment, occupation, BMI, status of smoking and drinking, and site and type of surgery.
Abbreviation: POCD: Postoperative cognitive dysfunction; OR: Odds Ratio, CI: confidence interval, GAD-7: Generalized Anxiety Disorder 7-item, PHQ-9: Patient Health Questionnaire-9, PSQI: Pittsburgh sleep quality index; ASA: American Society of Anesthesiologists, ICU: Intensive care unit

© 2023 Yang L et al. *JAMA Network Open.*

eTable 8 Subgroup analysis of factors associated with short-term memory (measured by Three-word Recall Test [TRT] score) impairment among participants receiving noncardiac surgery

Variables	By sex			By age		
	Male OR (95% CI)	Female OR (95% CI)	p for interaction	40-55 years OR (95% CI)	55-65 years OR (95% CI)	p for interaction
Comorbidity (yes vs no)						
Psychiatric disorder	1.09 (0.59-2.02)	1.04 (0.77-1.40)	0.8923	1.05 (0.77-1.44)	0.96 (0.57-1.59)	0.7995
Diabetes	1.04 (0.87-1.25)	1.24 (0.96-1.61)	0.3100	1.16 (0.92-1.45)	1.08 (0.88-1.32)	0.6796
Hepatic disease	1.19 (1.02-1.39)	0.96 (0.83-1.11)	0.0482	1.07 (0.93-1.21)	1.02 (0.86-1.23)	0.7715
Peptic ulcer	1.38 (1.10-1.74)	1.30 (0.99-1.71)	0.8385	1.11 (0.87-1.42)	1.68 (1.29-2.18)	0.0209
Solid tumor	1.19 (0.87-1.63)	1.05 (0.84-1.32)	0.5204	1.21 (0.96-1.54)	0.98 (0.74-1.29)	0.2509
Chronic pain [c]	1.08 (0.93-1.26)	1.04 (0.93-1.16)	0.7545	1.07 (0.96-1.20)	1.02 (0.87-1.19)	0.6853
Pain in the week before surgery	1.20 (0.96-1.49)	1.00 (0.85-1.18)	0.1859	1.08 (0.93-1.26)	1.03 (0.80-1.33)	0.7499
Psychological condition						
Anxiety (GAD-7 score ≥5 vs <5)	0.91 (0.67-1.24)	1.15 (0.98-1.34)	0.1526	1.04 (0.89-1.21)	1.30 (0.95-1.79)	0.2197
Depression (PHQ-9 score ≥5 vs <5)	1.19 (0.96-1.48)	1.16 (1.01-1.33)	0.9276	1.21 (1.05-1.39)	1.06 (0.85-1.31)	0.2967
Sleep disturbance (PSQI score)						
0-5	Ref	Ref		Ref	Ref	
6-10	1.17 (1.04-1.33)	1.22 (1.10-1.34)	0.6754	1.22 (1.11-1.34)	1.13 (0.99-1.29)	0.363
11-15	1.34 (1.06-1.68)	1.32 (1.14-1.52)	0.8463	1.31 (1.12-1.53)	1.26 (1.03-1.54)	0.7095
≥16	1.26 (0.58-2.71)	1.45 (0.93-2.26)	0.7579	1.79 (1.08-2.98)	0.97 (0.54-1.73)	0.0927
Anesthesia/Surgery-related						
Site of surgery						
Head and neck	Ref	Ref		Ref	Ref	
Thorax	0.96 (0.78-1.18)	1.00 (0.85-1.18)	0.6807	0.93 (0.80-1.09)	1.11 (0.88-1.40)	0.2041
Abdomen	1.00 (0.86-1.17)	1.11 (0.97-1.28)	0.2558	1.03 (0.91-1.16)	1.17 (0.97-1.42)	0.2759
Limbs and surface	0.98 (0.72-1.33)	0.85 (0.83-1.09)	0.9791	0.94 (0.82-1.08)	1.02 (0.80-1.31)	0.5606
Other	1.12 (0.84-1.49)	1.24 (0.97-1.58)	0.5807	1.13 (0.90-1.41)	1.28 (0.92-1.79)	0.4752
Type of surgery (Open vs Endoscopic)	1.04 (0.92-1.18)	1.10 (0.98-1.23)	0.54	1.01 (0.91-1.11)	1.20 (1.03-1.39)	0.0696
Anesthesia duration (> 3 hours vs ≤ 3 hours)	1.12 (0.98-1.28)	1.09 (0.97-1.23)	0.7332	1.05 (0.94-1.17)	1.24 (1.06-1.44)	0.0881
ASA grade (≥3 vs <3)	1.13 (0.95-1.34)	1.20 (0.99-1.46)	0.572	1.21 (1.01-1.46)	1.11 (0.92-1.34)	0.4586
Type of general anesthesia maintenance						
Total intravenous anesthesia	Ref	Ref		Ref	Ref	
Combined intravenous and inhalation anesthesia	0.94 (0.68-1.29)	1.18 (0.96-1.45)	0.2249	1.04 (0.85-1.29)	1.24 (0.91-1.68)	0.2788
Inhalation anesthesia	1.05 (0.58-1.91)	1.13 (0.71-1.79)	0.829	1.13 (0.72-1.79)	1.14 (0.63-2.07)	0.8480
Nerve block (yes vs no)	1.06 (0.93-1.20)	0.99 (0.89-1.10)	0.393	1.00 (0.90-1.11)	1.04 (0.91-1.19)	0.6875
Blood transfusion (yes vs no)	1.20 (0.90-1.60)	1.49 (1.12-1.98)	0.2701	1.28 (0.98-1.67)	1.45 (1.05-1.99)	0.5281
Severe hypoxia [a] (yes vs no)	1.32 (1.03-1.70)	1.12 (0.90-1.38)	0.3569	1.22 (0.98-1.51)	1.20 (0.93-1.54)	0.8737
Severe hypotension [b] (yes vs no)	1.01 (0.88-1.18)	1.01 (0.90-1.14)	0.9627	1.04 (0.93-1.17)	0.97 (0.84-1.13)	0.4660

Continued on next page

© 2023 Yang L et al. *JAMA Network Open.*

Variables	By sex: Male OR (95% CI)	By sex: Female OR (95% CI)	By sex: p for interaction	By age: 40-55 years OR (95% CI)	By age: 55-65 years OR (95% CI)	By age: p for interaction
**Postsurgical events**						
Patient-controlled analgesia (yes vs no)	1.18 (1.03-1.35)	1.12 (0.99-1.27)	0.6539	1.15 (1.02-1.29)	1.14 (0.98-1.33)	0.9478
Admission to ICU (yes vs no)	1.25 (0.90-1.75)	1.65 (1.06-2.56)	0.2843	1.25 (0.91-1.72)	1.63 (0.99-2.70)	0.0544
Acute postoperative pain (BPI ≥ 4 vs <4 at 3 days)	1.03 (0.75-1.42)	1.49 (1.20-1.86)	0.2271	1.36 (1.09-1.70)	1.27 (0.94-1.71)	0.4283
Any Postoperative complications [c] (yes vs no)	1.24 (1.05-1.46)	1.07 (0.90-1.28)	0.0558	1.09 (0.93-1.27)	1.22 (1.01-1.47)	0.6962

[a] SpO2 < 90% during the surgery;
[b] Atrial pressure < 60% of baseline level during the surgery;
[c] Included pulmonary complication, major adverse cardiac event, acute kidney injury, and infection.
OR and 95%CI were derived from generalized liner mixed-effect models, adjusted for age, sex, ethnicity, educational attainment, occupation, BMI, status of smoking and drinking, and site and type of surgery.
Abbreviation: POCD: Postoperative cognitive dysfunction; OR: Odds Ratio, CI: confidence interval, GAD-7: Generalized Anxiety Disorder 7-item, PHQ-9: Patient Health Questionnaire-9, PSQI: Pittsburgh sleep quality index; ASA: American Society of Anesthesiologists, ICU: Intensive care unit

© 2023 Yang L et al. *JAMA Network Open.*

eTable 9 Sensitivity analysis of factors associated with aggressively deteriorative trajectory of 8-item Interview to Differentiate Aging and Dementia [AD-8] scores and Three-word Recall Test [TRT] scores by using the ‘consistently low-risk trajectory’ as reference group

Variables	Participants received non-cardiac surgery		Participants received cardiac surgery
	Aggressively deteriorative trajectory of AD-8 scores	Aggressively deteriorative trajectory of TRT scores	Aggressively deteriorative trajectory of AD-8 scores
	OR (95% CI)	OR (95% CI)	OR (95% CI)
Comorbidity (yes vs no)			
Psychiatric disorder	2.05 (1.24-3.39)	1.62 (0.80-3.25)	2.14 (0.21-21.49)
Diabetes	1.01 (0.73-1.39)	0.91 (0.61-1.36)	0.55 (0.12-2.45)
Hepatic disease	1.28 (1.01-1.61)	1.05 (0.79-1.41)	1.48 (0.66-3.29)
Peptic ulcer	1.27 (0.87-1.85)	1.51 (0.96-2.38)	2.66 (0.99-7.20)
Solid tumor	1.23 (0.85-1.77)	1.01 (0.62-1.65)	0.00 (0.00-Inf)
Preoperative pain	1.26 (1.03-1.54)	1.11 (0.87-1.42)	1.78 (0.88-3.62)
Pain in the week before surgery	1.51 (1.16-1.97)	0.82 (0.56-1.22)	1.69 (0.81-3.54)
Psychological condition			
Anxiety (GAD-7 score ≥5 vs <5)	1.47 (1.06-2.04)	1.35 (0.91-2.02)	1.78 (0.65-4.83)
Depression (PHQ-9 score ≥5 vs <5)	1.93 (1.51-2.46)	1.12 (0.81-1.56)	2.01 (0.89-4.56)
Sleep disturbance (PSQI score)			
0-5	Ref	Ref	Ref
6-10	1.49 (1.25-1.77)	1.21 (0.98-1.51)	1.60 (0.86-2.98)
11-15	2.04 (1.59-2.61)	1.90 (1.40-2.57)	0.64 (0.20-2.01)
≥ 16	4.30 (2.31-8.01)	0.00 (0.00-Inf)	4.52 (0.78-26.11)
Anesthesia/Surgery-related			
Site of surgery			
Head and neck	Ref	Ref	-
Thorax	1.34 (0.98-1.83)	0.72 (0.49-1.05)	-
Abdomen	1.66 (1.30-2.12)	0.97 (0.73-1.28)	-
Limbs and surface	1.43 (1.08-1.88)	0.76 (0.54-1.08)	-
Other	0.58 (0.35-0.97)	0.64 (0.38-1.09)	-
Type of surgery (Open vs Endoscopic)	1.34 (1.11-1.62)	1.06 (0.84-1.34)	0.61 (0.12-2.97)
Anesthesia duration [a] (> 3 hours vs ≤ 3 hours)	1.36 (1.13-1.63)	1.13 (0.89-1.44)	1.05 (0.50-2.19)
ASA grade (≥3 vs <3)	1.13 (0.85-1.49)	1.33 (0.96-1.82)	0.37 (0.09-1.44)
Type of general anesthesia maintenance			
Total intravenous anesthesia	Ref	Ref	Ref
Combined intravenous and inhalation anesthesia	0.77 (0.53-1.11)	0.83 (0.52-1.30)	0.39 (0.14-1.11)
Inhalation anesthesia	1.75 (0.89-3.44)	1.43 (0.59-3.47)	0.00 (0.00-Inf)
Nerve block (yes vs no)	1.08 (0.90-1.29)	1.24 (0.99-1.56)	0.43 (0.05-3.35)
Blood transfusion (yes vs no)	1.02 (0.67-1.55)	1.11 (0.65-1.89)	2.21 (0.76-6.41)
Severe hypoxia [b],(yes vs no)	0.73 (0.50-1.05)	1.27 (0.85-1.90)	1.98 (1.05-3.75)
Severe hypotension [c], (yes vs no)	0.88 (0.72-1.08)	1.09 (0.85-1.40)	1.66 (0.66-4.13)
**Postsurgical events**			
Patient-controlled analgesia (yes vs no)	1.24 (1.02-1.52)	1.27 (0.99-1.63)	0.00 (0.00-Inf)

Continued on next page

© 2023 Yang L et al. *JAMA Network Open.*

Variables	Participants received non-cardiac surgery		Participants received cardiac surgery
	Aggressively deteriorative trajectory of AD-8 scores	Aggressively deteriorative trajectory of TRT scores	Aggressively deteriorative trajectory of AD-8 scores
	OR (95% CI)	OR (95% CI)	OR (95% CI)
Admission to ICU (yes vs no)	2.04 (1.23-3.37)	1.73 (0.88-3.41)	1.48 (0.18-12.38)
Length of ICU stay, d			
0 day	Ref	Ref	Ref
1 day	1.30 (0.58-2.90)	1.06 (0.36-3.14)	1.12 (0.13-10.33)
≥ 2 days	3.05 (1.55-5.99)	2.28 (0.85-6.14)	2.17 (0.27-17.40)
Acute postoperative pain (BPI ≥ 4 vs <4 at 3 days)	1.37 (0.97-1.93)	1.08 (0.65-1.82)	0.50 (0.11-2.26)
Any Postoperative complications [d] (yes vs no)	1.27 (1.00-1.63)	1.02 (0.74-1.41)	1.17 (0.64-2.12)

OR and 95%CI were derived from logistic regression models, adjusted for age, sex, ethnicity, educational attainment, occupation, BMI, status of smoking and drinking, and site and type of surgery.

[a] The corresponding anesthesia duration categories among participants receiving cardiac surgery were ≤ 6 hours, >6 hours respectively

[b] SpO2 < 90% during the surgery;

[c] Atrial pressure < 60% of baseline level during the surgery;

[d] Included pulmonary complication, major adverse cardiac event, acute kidney injury, and infection.

Abbreviation: OR: Odds Ratio, CI: confidence interval, GAD-7: Generalized Anxiety Disorder 7-item, PHQ-9: Patient Health Questionnaire-9, PSQI: Pittsburgh sleep quality index; ASA: American Society of Anesthesiologists, ICU: Intensive care unit,

© 2023 Yang L et al. *JAMA Network Open.*
