# Supplementary material for: Postsurgery Subjective Cognitive and Short-Term Memory Impairment Among Middle-Aged Chinese Patients
Source: JAMA Netw Open. 2023 Oct 10;6(10):e2336985. doi: 10.1001/jamanetworkopen.2023.36985 (PMC10565601; doi:10.1001/jamanetworkopen.2023.36985)
Supplement: Supplement 2. — Data Sharing Statement [file jamanetwopen-e2336985-s002.pdf]

Data Sharing Statement

Yang. Postsurgery Subjective Cognitive and Short-Term Memory Impairment Among Middle-Aged Chinese Patients. *JAMA Netw Open*. Published October 11, 2023. doi:10.1001/jamanetworkopen.2023.36985

Data
Data available: No
